# Treatment patterns and characteristics of patients with Post-Traumatic Stress Disorder (PTSD): A retrospective claims analysis among commercially insured population

**DOI:** 10.1371/journal.pone.0309704

**Published:** 2024-10-30

**Authors:** Filip Stanicic, Vladimir Zah, Dimitrije Grbic, Debra De Angelo, Wendy Bibeau

**Affiliations:** 1 ZRx Outcomes Research Inc., Mississauga, ON, Canada; 2 Lykos Therapeutics, San Jose, CA, United States of America; Taipei Veterans General Hospital, TAIWAN

## Abstract

**Objective:**

This retrospective claims analysis explored the treatment utilization and characteristics among patients with post-traumatic stress disorder (PTSD) of different severity.

**Methods:**

The index date was the first PTSD claim. The analysis observed 12 months pre- and 24 months post-index. Adults with insurance gaps, cancer, or acute PTSD during the observation were excluded. Patients were categorized into three severity cohorts based on treatment and healthcare services utilization for PTSD: 1. Baseline PTSD (BP) (no PTSD visits post-index, no FDA-approved medications/ psychotherapy, and no severe mental health comorbidities); 2. PTSD without Comorbidities (PwoC) (≥1 PTSD visits post-index and no severe mental health conditions); 3. PTSD with Comorbidities (PwC) (≥1 PTSD visits post-index and severe mental health comorbidities present). For the primary analysis, cohorts were propensity-score matched. A sub-analysis examined patients with PTSD and Substance or Alcohol Use Disorder (SUD/AUD).

**Results:**

The primary analysis observed 1714 BP, 1681 PwoC, and 1681 PwC patients. Treatment utilization rates were highest among PwC vs. other cohorts (84.5% psychotherapy, 76.1% off-label medications, and 26.1% FDA-approved medications [p<0.001]). PwC cohort also had the highest number of psychotherapy sessions and medication prescriptions per patient (20.1 sessions, 12.6 off-label prescriptions, and 2.0 FDA-approved prescriptions [p<0.001]). The proportion of days covered (PDC) indicated low medication adherence (0.25–0.40) with adherent patient rates (PDC ≥0.80) between 8.0–17.5%. The SUD/AUD sub-analysis identified 85 BP, 537 PwoC, and 3154 PwC patients. Conclusions were similar, with PwC cohort having highest treatment utilization rates (87.1% psychotherapy, 85.0% off-label medications, 28.2% FDA-approved medications [p≤0.013] with 24.4 sessions, 16.1 off-label prescriptions, and 2.0 FDA-approved prescriptions per patient [p≤0.002]). Only 4.7–11.4% of patients were adherent.

**Conclusions:**

PwC patients received psychotherapy and pharmacotherapy more frequently than PwoC and BP patients. Medication adherence among treated patients was low. Patients with SUD/AUD had numerically higher treatment utilization and lower medication adherence.

## Introduction

Post-traumatic stress disorder (PTSD) is a disorder characterized by symptoms of re-experiencing memories of a traumatic event, avoidance, negative alterations in cognition and mood, and marked alterations in arousal and reactivity following exposure to trauma [1]. Symptoms of PTSD include distressing and intrusive memories, and nightmares of trauma, irritability, hypervigilance, difficulty sleeping, poor concentration, and emotional withdrawal. Individuals with PTSD frequently avoid places, activities, or things that could remind them of the traumatic event [2].

The estimated 1-year PTSD prevalence is 2.3–9.1% and lifetime prevalence 3.4–26.9% among the general US population, with a 2-fold higher prevalence among women than men [3]. Different experiences may lead to PTSD diagnosis. PTSD occurrence is estimated to be higher in the military population, with a 1-year prevalence ranging from 6.7–50.2% and a lifetime prevalence of 7.7–17.0% [3]. Lifetime PTSD prevalence is estimated to be 13.4% among female veterans and 7.7% in male veterans [4]. Still, non-combat-related trauma represents the most frequent cause of PTSD (childhood trauma, sexual and interpersonal violence, traumatic event in social or family networks, etc.) [5].

The excess economic burden of PTSD in the US was estimated to be $19,630 per individual diagnosed with PTSD and $232.2 billion for the US healthcare system in 2018 [6]. Specifically, total excess costs of PTSD were $189.5 billion in the civilian and $42.7 billion in the military population, with corresponding costs of $18,640 and $25,684 per individual with PTSD in the civilian and military populations. The excess PTSD burden was mostly driven by direct health care and unemployment in the civilian population and by disability and direct health care in the military population [6].

Based on the most recent guidelines from the American Psychological Association (APA) and Veterans Affairs/Department of Defense (VA/DoD), there are limited treatment options for PTSD management. APA clinical practice guidelines for the treatment of PTSD in adults (2017) provide moderate recommendations for selective serotonin reuptake inhibitors (SSRIs) and serotonin and norepinephrine reuptake inhibitors (SNRIs) usage in the management of PTSD [7]. VA/DoD clinical practice guidelines (2023) provide strong recommendations for pharmacotherapy with SSRIs (paroxetine and sertraline) and SNRI (venlafaxine) [8]. Other pharmacological treatments do not receive strong recommendations for PTSD management due to evidence inconsistencies related to efficacy and safety. Besides pharmacotherapy, both guidelines provide strong evidence for individual, manualized trauma-focused psychotherapy treatments for patients with PTSD [7].

PTSD is commonly associated with substance use disorders [9]. Based on the National Comorbidity Survey, respondents with PTSD were three times as likely to report drug abuse or dependence and twice as likely to report alcohol use disorder (AUD) [10, 11]. The complex and highly burdensome association of PTSD and substance use disorders (SUD) requires a more intensive treatment approach with integrated, sequential psychosocial and pharmacological therapeutic interventions and potential treatment augmentation [9].

This study aimed to explore treatment characteristics and adherence to pharmacological treatment among patients with chronic PTSD. The primary analysis assessed study outcomes among a total sample of patients with PTSD, while an additional sub-analysis evaluated outcome measures within patients with PTSD and diagnosed SUD or AUD.

## Materials and methods

The research was performed in accordance with the Strengthening the Reporting of Observational Studies in Epidemiology (STROBE) cohort studies recommendations [12].

### Data source

The retrospective cohort study was conducted using US insurance claims data from the Merative MarketScan® Commercial and Medicare Part B claims database. This database consisted of medical and prescription data for over 293 million individuals, encompassing employees, their spouses, and dependents who are covered by employer-sponsored private health insurance in the US [13]. The database complies with the Health Insurance Portability and Accountability Act of 1996 (HIPAA), protecting patient’s privacy and ensuring the confidentiality of personal data. In addition, only de-identified data was used in the study, whereby approval of Institutional Review Board for conducting the research was not required. This study evaluated insurance data claims captured between November 01, 2017 and October 31, 2022. The authors had accessed to the database starting from 29/06/2023.

### Study population

Pre-defined inclusion/exclusion criteria were employed to identify the most relevant sample of patients with chronic PTSD included in the primary analysis.

Inclusion criteria were adult patients (≥18 years) diagnosed with PTSD based on the International Classification of Diseases–Clinical Modification (ICD-10-CM) codes F43.1, F43.10, and F43.12 (S1 Table in S1 File). To avoid selection bias, patients with only acute PTSD claims (F43.11) (S2 Table in S1 File) were excluded as symptoms, in this case, last for less than three months and require less intensive treatment [14]. Other exclusion criteria were a gap in healthcare or pharmaceutical coverage, and a cancer diagnosis (S3 Table in S1 File) during the observational period (including 12-month pre-index and 24-month post-index period).

The sub-analysis population was selected by applying an additional inclusion criterion—patients with chronic PTSD and diagnosed SUD/AUD based on ICD-10-CM codes (S4 Table in S1 File) during the observational period.

### Study design

**Fig 1:**
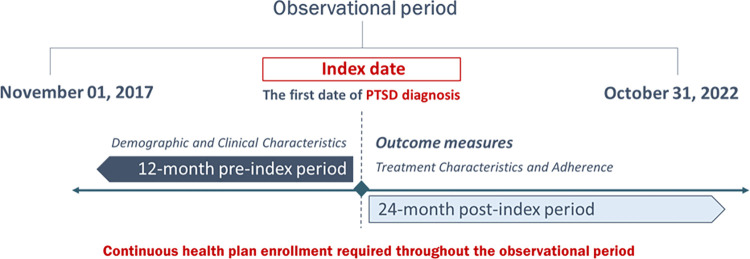
Study design.

This was an observational, retrospective, claims database analysis. Patients diagnosed with chronic PTSD were captured in the database from November 01, 2017 to October 31, 2022. The first PTSD diagnosis was deemed to be the index date, and the study observational period consisted of 12-month pre- and 24-month post-index periods. Patients were required to have continuous healthcare and pharmaceutical coverage during the whole observational period. Based on the number of PTSD visits in follow-up and a proxy for disease severity, patients were assigned to one of three study cohorts: Baseline PTSD (BP), PTSD without Comorbidities (PwoC), and PTSD with Comorbidities (PwC). Patients with no PTSD visits in the post-index period apart from the baseline PTSD visit (index claim) and without a diagnosis of major depressive disorder (MDD), bipolar disorder, or schizophrenia during the 24-month post-index period were assigned to the BP cohort. In addition, BP patients did not receive US Food and Drug (FDA)-approved medications or psychotherapy during the observational period. Patients with more than one PTSD visit (other than the PTSD claim on the index date) were stratified into PwoC and PwC cohorts. PwoC did not include patients with MDD, bipolar disorder, or schizophrenia claims during the 24-month post-index period, while patients with these mental health comorbidities during the follow-up period were categorized as PwC [15, 16]. MDD, bipolar disorder, and schizophrenia were captured based on the relevant ICD-10-CM codes (S5 Table in S1 File). Currently, the only FDA-approved medicines for PTSD treatment are sertraline and paroxetine. All other pharmacotherapeutics are used off-label, with varying level of evidence supporting their administration. The analysis considered these two major groups of pharmacotherapies in order to observe the frequency of their use in clinical practice, as well as to analyze the difference in expenditures emerging with their use. FDA-approved medications for PTSD treatment were identified via relevant National Drug Code (NDC) codes (S6 Table in S1 File), while psychotherapy was captured using Current Procedural Terminology (CPT) codes (S7 Table in S1 File). The study design is presented in Fig 1.

Demographic characteristics of the study population were assessed on the index date, while clinical characteristics were evaluated during the 12-month pre-index period. Treatment characteristics and adherence measures were assessed during the 24-month post-index period.

### Outcome measures

Primary analysis included all patients with chronic PTSD, while sub-analysis focused on the chronic PTSD population with comorbid SUD/AUD. All outcome measures were estimated in both primary and sub-analysis.

### Treatment characteristics

The proportions of patients treated with FDA-approved medications for PTSD treatment, off-label PTSD medications, and psychotherapy were evaluated. Off-label medications were identified based on the latest VA/DoD clinical practice guidelines [8] and relevant publicly available literature. The following list of off-label medications was assessed in the analysis: fluoxetine, venlafaxine, escitalopram, nefazodone, imipramine, amitriptyline, mirtazapine, phenelzine, bupropion, quetiapine, olanzapine, topiramate, lamotrigine, buspirone, citalopram, desvenlafaxine, eszopiclone, prazosin, pregabalin, rivastigmine, and duloxetine. The outcome measures were reported for the top 5 most frequently utilized off-label medications. The numbers of FDA-approved and off-label medication prescriptions were also explored. In addition, time-to-first psychotherapy, time between two psychotherapy sessions, and the number of psychotherapy sessions during the follow-up period were observed. PTSD treatment-related healthcare costs (FDA-approved PTSD medications, off-label PTSD medications, and psychotherapy sessions) were evaluated from the payer perspective (including only payer-related costs) and as the total healthcare costs (including both patients’ out-of-pocket and payer-related costs). The mean cost of psychotherapy session and total PTSD medication treatment costs were reported.

### Adherence to pharmacological treatment

**Fig 2:**
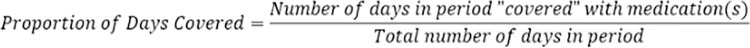
The formula for PDC calculation.

Medication adherence was evaluated using the proportion of days covered (PDC). The following formula (Fig 2) was employed to calculate PDC.

The PDC value was estimated as the sum of days a patient had access to medication divided by the total number of days in the follow-up period. The PDC considered whether patients were covered with the medication or not, regardless of the potential prescription overlap, therefore; the PDC value cannot exceed a score of 1.00. It was assumed that patients were adherent to the specific PTSD treatment if the PDC value was higher than a 0.80 threshold.

### Statistical analysis

Continuous variables were summarized as means with standard deviation, while categorical variables were summarized as numbers and proportions of the sample.

One-way ANOVA with Tamhane’s T2 post-hoc test was employed to test the differences between three study cohorts for continuous variables, while an independent t-test was performed to test the difference between two comparable cohorts. The chi-square test of independence was performed to test the differences related to the categorical variables. P-values lower than 0.05 implied a statistical significance occurred between the cohorts.

Propensity-score matching (PSM) with the nearest-neighbor matching algorithm was performed to balance the cohorts and minimize selection bias. The demographic and clinical characteristics of patients were utilized for the matching process. PSM in a 1:1:1 ratio was employed to match BP, PwoC, and PwC cohorts based on cohort size (the smallest cohort was matched with the middle cohort in a 1:1 ratio, followed by matching of the middle-sized cohort with the largest cohort in a 1:1 ratio).

## Results

### Primary analysis

**Fig 3:**
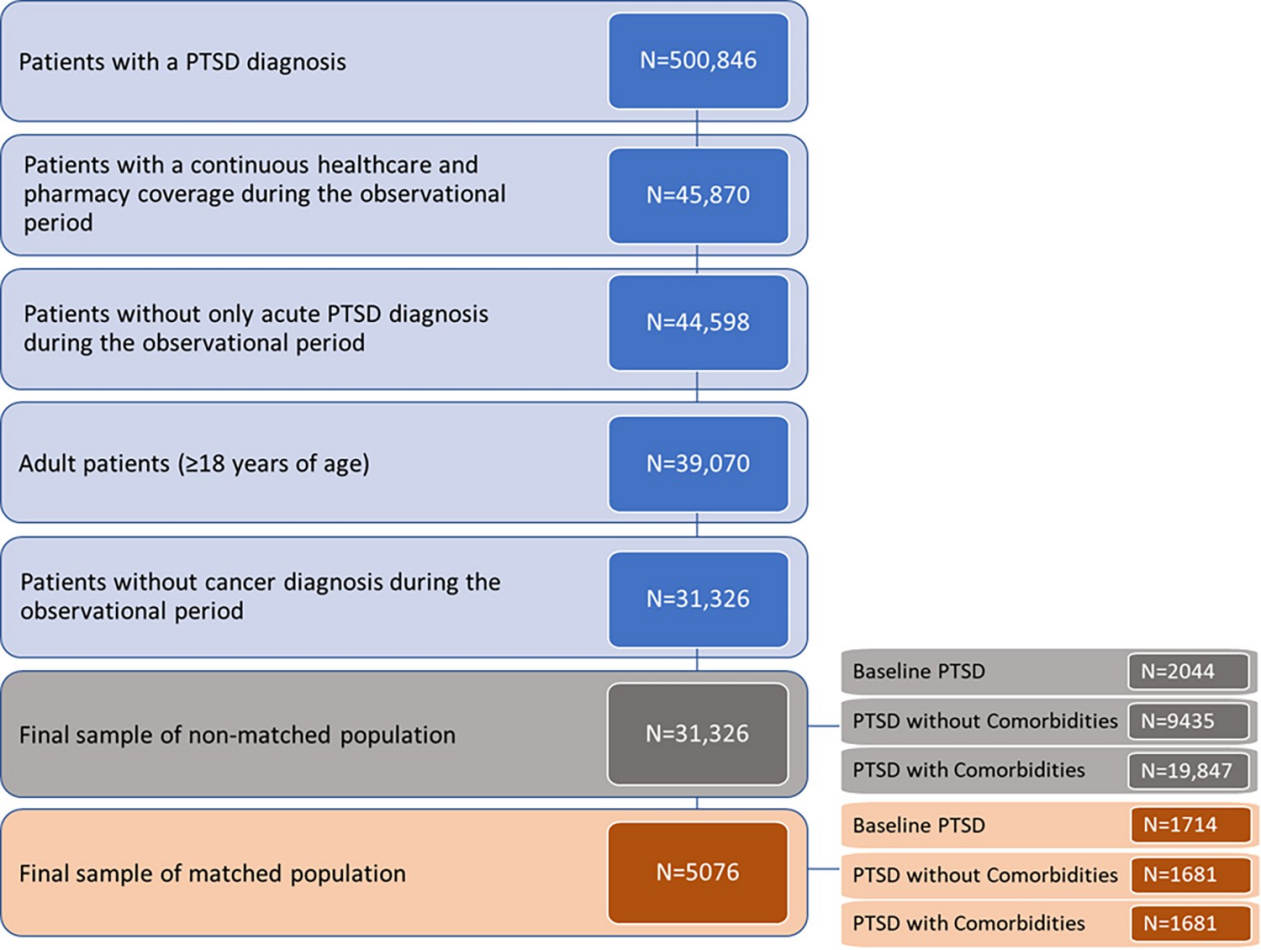
Patient selection flow diagram.

After applying patient selection criteria to 500,846 patients with a PTSD diagnosis, 31,326 were selected in the final sample of the non-matched population (2044 BP, 9435 PwoC, and 19,847 PwC). After the PSM analysis, the final sample of matched patients consisted of 5076 patients (1714 BP, 1681 PwoC, and 1681 PwC). The patient selection flow diagram is presented in Fig 3.

### Non-matched population

The mean age of patients was 38.4 years, with BP patients being older than patients without and with comorbidities (42.0 vs. 39.4 and 37.6 years, respectively; both p<0.001). The proportion of women was 70.0% in the total sample, with the highest prevalence in the PwC compared to the PwoC and BP cohorts (74.6% vs. 64.4% and 50.5%, respectively; both p<0.001). Different types of health insurance plans in the US are designed to meet patients’ needs and budgets. Besides the monthly premium, the key differences between plans include flexibility when choosing healthcare providers and expenditures for deductibles and copayments [17]. Most patients with PTSD were covered by Preferred Provider Organization (49.1%) and Health Maintenance Organization (18.0%) insurance plans. These are common health insurance plans in the US, characterized by a low level of freedom to choose the healthcare provider and high out-of-pocket costs for services outside of the provider’s network [18]. All demographic characteristics are presented in Table 1.

**Table 1:** Demographic characteristics of the non-matched population.

	Total sample (N = 31,326)	Baseline PTSD (N = 2044)	PTSD without Comorbidities (N = 9435)	PTSD with Comorbidities (N = 19,847)	P-value*
Age, mean (SD)	38.4 (12.8)	42.0 (12.2)	39.4 (12.3)	37.6 (13.0)	**<0.001** ^**1**^^,^ ^**2**^^,^ ^**3**^
**Gender, n (%)**		** **	** **	** **	** **
Women	21,918 (70.0)	1032 (50.5)	6076 (64.4)	14,810 (74.6)	**<0.001** ^**1**^^,^ ^**2**^^,^ ^**3**^
Men	9408 (30.0)	1012 (49.5)	3359 (35.6)	5037 (25.4)	**<0.001** ^**1**^^,^ ^**2**^^,^ ^**3**^
**Health Plan, n (%)**		** **	** **	** **	** **
Preferred Provider Organization	15,376 (49.1)	1003 (49.1)	4612 (48.9)	9761 (49.2)	0.892
Health Maintenance Organization	5648 (18.0)	398 (19.5)	1693 (17.9)	3557 (17.9)	0.215
Consumer-Driven Health Plan	3958 (12.6)	246 (12.0)	1136 (12.0)	2576 (13.0)	**0.024** ^**3**^
High-Deductible Health Plan	3357 (10.7)	217 (10.6)	1084 (11.5)	2056 (10.4)	**0.003** ^**3**^
Non-Capitated Point-of-Service	1567 (5.0)	91 (4.5)	521 (5.5)	955 (4.8)	**0.009** ^**3**^
Comprehensive Plan	920 (2.9)	61 (3.0)	240 (2.5)	619 (3.1)	**0.006** ^**3**^
Unknown	290 (0.9)	14 (0.7)	84 (0.9)	192 (1.0)	0.407
Exclusive Provider Organization	134 (0.4)	9 (0.4)	44 (0.5)	81 (0.4)	0.772
Point-of-Service with Capitation	76 (0.2)	5 (0.2)	21 (0.2)	50 (0.3)	0.892
**Region, n (%)**		** **	** **	** **	** **
South	13,040 (41.6)	984 (48.1)	3743 (39.7)	8313 (41.9)	**<0.001** ^**1**^^,^ ^**2**^^,^ ^**3**^
North Central	7409 (23.7)	387 (18.9)	2162 (22.9)	4860 (24.5)	**≤0.003** ^**1**^^,^ ^**2**^^,^ ^**3**^
West	6759 (21.6)	445 (21.8)	2156 (22.9)	4158 (21.0)	**<0.001** ^**3**^
Northeast	4072 (13.0)	226 (11.1)	1354 (14.4)	2492 (12.6)	**<0.001** ^**1**^^,^^**3**^
Unknown	46 (0.1)	2 (0.1)	20 (0.2)	24 (0.1)	0.137

*Chi-square test was performed for categorical variables and ANOVA (Tamhane’s T2 test) for continuous variables

^1^ Baseline PTSD vs. PTSD without Comorbidities, p<0.05

^2^ Baseline PTSD vs. PTSD with Comorbidities, p<0.05

^3^ PTSD without Comorbidities vs. PTSD with Comorbidities, p<0.05

Charlson Comorbidity Index (CCI) was employed to assess pre-index clinical characteristics of patients (Table 2). It was demonstrated that 73.9% of patients with PTSD were stratified in the CCI = 0 category, while the mean CCI score was 0.5 points. The mean CCI scores in PwC (0.5) and BP (0.5) were significantly higher than in PwoC (0.4, both p<0.001). Significant differences between study cohorts were demonstrated in almost all CCI components except for moderate or severe liver disease (p = 0.369).

**Table 2:** Clinical characteristics of the non-matched population.

	Total sample (N = 31,326)	Baseline PTSD (N = 2044)	PTSD without Comorbidities (N = 9435)	PTSD with Comorbidities (N = 19,847)	P-value*
**Charlson Comorbidity Index, n (%)**					
0	23,151 (73.9)	1521 (74.4)	7362 (78.0)	14,268 (71.9)	**≤0.015** ^**1**^^,^ ^**2**^^,^ ^**3**^
1	4729 (15.1)	295 (14.4)	1252 (13.3)	3182 (16.0)	**≤0.007** ^**3**^
2	1477 (4.7)	91 (4.5)	352 (3.7)	1034 (5.2)	**<0.001** ^**3**^
3	1136 (3.6)	62 (3.0)	307 (3.3)	767 (3.9)	**0.009** ^**3**^
4+	833 (2.7)	75 (3.7)	162 (1.7)	596 (3.0)	**<0.001** ^**1**^^,^ ^**3**^
Charlson Comorbidity Index, mean (SD)	0.5 (1.1)	0.5 (1.2)	0.4 (0.9)	0.5 (1.1)	**<0.001** ^**1**^^,^ ^**3**^
**Charlson Comorbidity Index Components, n (%)**					
Chronic pulmonary disease	3449 (11.0)	187 (9.1)	851 (9.0)	2411 (12.1)	**<0.001** ^**2**^^,^ ^**3**^
Diabetes without chronic complication	2273 (7.3)	173 (8.5)	572 (6.1)	1528 (7.7)	**<0.001** ^**1**^^,^ ^**3**^
Diabetes with chronic complication	1353 (4.3)	104 (5.1)	327 (3.5)	922 (4.6)	**<0.001** ^**1**^^,^ ^**3**^
Renal disease	1233 (3.9)	92 (4.5)	286 (3.0)	855 (4.3)	**≤0.001** ^**1**^^,^ ^**3**^
Mild liver disease	1160 (3.7)	82 (4.0)	288 (3.1)	790 (4.0)	**≤0.026** ^**1**^^,^ ^**3**^
Cerebrovascular disease	588 (1.9)	38 (1.9)	151 (1.6)	399 (2.0)	**0.016** ^**3**^
Rheumatic disease	556 (1.8)	25 (1.2)	123 (1.3)	408 (2.1)	**≤0.010** ^**2**^^,^ ^**3**^
Peripheral vascular disease	270 (0.9)	26 (1.3)	65 (0.7)	179 (0.9)	**0.007** ^**1**^
Congestive heart failure	253 (0.8)	25 (1.2)	64 (0.7)	164 (0.8)	**0.011** ^**1**^
Peptic ulcer disease	197 (0.6)	17 (0.8)	33 (0.3)	147 (0.7)	**≤0.003** ^**1**^^,^ ^**3**^
Hemiplegia or paraplegia	142 (0.5)	4 (0.2)	29 (0.3)	109 (0.5)	**≤0.033** ^**2**^^,^ ^**3**^
Myocardial infarction	165 (0.5)	19 (0.9)	42 (0.4)	104 (0.5)	**≤0.020** ^**1**^^,^ ^**2**^
AIDS/HIV	127 (0.4)	8 (0.4)	26 (0.3)	93 (0.5)	**0.015** ^**3**^
Dementia	79 (0.3)	4 (0.2)	16 (0.2)	59 (0.3)	**0.043** ^**3**^
Moderate or severe liver disease	50 (0.2)	1 (0.0)	14 (0.1)	35 (0.2)	0.369
Malignancy	0	0	0	0	-
Metastatic solid tumor	0	0	0	0	-

*Chi-square test was performed for categorical variables and ANOVA (Tamhane’s T2 test) for continuous variables

^1^ Baseline PTSD vs. PTSD without Comorbidities, p<0.05

^2^ Baseline PTSD vs. PTSD with Comorbidities, p<0.05

^3^ PTSD without Comorbidities vs. PTSD with Comorbidities, p<0.05

### Matched population

Demographic and clinical characteristics of the study population were used to match PTSD cohorts. Hence, there were no between-group differences observed in these characteristics. Matched patients were approximately 41 years of age, primarily women (53.7%), covered by Preferred Provider Organization insurance type (53.7%) and located in the South region (49.5%). Demographic characteristics of the matched study population are reported in S8 Table in S1 File. The mean CCI score was 0.3 and most patients had no relevant comorbidities (81.0%), with no between-group differences. Statistical significance was not detected in individual CCI comorbidities, with the highest rate of patients having chronic pulmonary disease (7.7%). All clinical characteristics are presented in S9 Table in S1 File.

During the 24-month follow-up period, 53.0% of patients were treated with PTSD medications, of which 12.9% of all patients were treated with FDA-approved and 48.5% with off-label. Significantly higher utilization rates and mean prescription counts of any PTSD medication were observed in the PwC cohort (82.9% of patients, mean 14.6 prescriptions per patient) compared to PwoC (45.5% of patients, mean 5.0 prescriptions; both p<0.001) and BP (31.0% of patients, mean 2.4 prescriptions; both p<0.001). The same trend was observed among patients receiving FDA-approved or off-label medications. The utilization rate of FDA-approved PTSD medications among patients with severe mental health comorbidities was 26.1% with mean 2.0 prescriptions per patient, while only 13.0% with a mean of 1.0 prescriptions was seen in patients without comorbidities (both p<0.001). Most patients with PTSD were prescribed sertraline (22.3% in PwC vs. 11.4% in PwoC, p<0.001).

For off-label PTSD medications, the utilization rate among patients with severe mental health comorbidities (76.1%) was significantly higher than in the PwoC and BP cohorts (38.7% and 31.0%, respectively; both p≤0.003). This trend was also observed for the number of off-label PTSD medication prescriptions (mean 12.6 prescriptions in PwC vs. 4.0 and 2.4 in the PwoC and BP cohorts, respectively; both p<0.001). The most frequently used off-label medication was bupropion (12.9%) with a higher utilization rate reported in the PwC cohort (25.5%) than among PwoC and BP patients (7.7% and 5.6%, respectively; both p≤0.013).

Treatment with off-label medications yielded payer and total costs of $343 and $460 per treated patient, respectively. Significantly higher costs were observed in the PwC cohort ($480 payer and $628 total costs) compared to the PwoC and BP cohorts ($226 and $157 payer, $319 and $227 total costs, respectively; all p≤0.015). Payer healthcare costs per treated patient associated with FDA-approved PTSD medications were $49 and total healthcare costs (payer and out-of-pocket) were $89 per patient.

Across the total study sample, psychotherapy utilization rates and the mean number of sessions per patient during the 24-month follow-up were 52.8% and 11.3 sessions, respectively. The results were significantly higher in the PwC cohort (84.5% and 20.1 sessions) than in the PwoC cohort (74.8% and 14.0 sessions, both p<0.001). Time to the first session from the index date was 50.9 days in PwC and 33.1 days in PwoC patients (p<0.001), while the mean gap between the two sessions was 19.1 days and 18.0 days, respectively (p = 0.005). The most common type of psychotherapy was individual session (83.9% of PwC and 74.3% of PwoC, p<0.001).

Mean healthcare cost associated with psychotherapy was $1950 per treated patient from the payer perspective, while the total cost (including payer and out-of-pocket) was $2501 per treated patient. Higher costs were reported in patients with severe mental health comorbidities for both payer ($2309 in PwC and $1545 in PwoC, p<0.001) and total costs ($2877 in PwC and $2077 PwoC, p<0.001). Healthcare costs related to psychotherapy interventions stratified by session duration and type are reported in S10 Table in S1 File.

Concomitant PTSD treatment, including psychotherapy and any PTSD pharmacological therapy, was observed in 34.0% of patients during the 24-month follow-up, while 28.3% of the total population did not receive any PTSD treatment relevant to this study. The proportion of PTSD patients with severe mental health comorbidities receiving any PTSD treatment (96.5%) was significantly higher than in the PwoC (88.5%, p<0.001) and BP cohorts (31.0%, p<0.001). PwC patients also more commonly received concomitant psychotherapy and PTSD medication treatment (70.8%) than PwoC patients (31.8%, p<0.001). All treatment characteristics of the total study sample and across PTSD cohorts are presented in Table 3.

**Table 3:** Treatment characteristics of the matched patients with PTSD during the 24-month post-index period.

	Total Sample (N = 5076)	Baseline PTSD (N = 1714)	PTSD without Comorbidities (N = 1681)	PTSD with Comorbidities (N = 1681)	P-value*
**FDA-Approved Medications, n (%)**	**656 (12.9)**	**0**	**218 (13.0)**	**438 (26.1)**	**<0.001** ^**1, 2, 3**^
Number of medications per patient, mean (SD) ^#^	1.0 (3.5)	0	1.0 (3.6)	2.0 (4.7)	**<0.001** ^**1, 2, 3**^
Number of medications per patient, mean (SD) †	7.6 (6.6)	-	7.6 (6.9)	7.6 (6.5)	0.949
Cost per patient from payer perspective, mean (SD) †	$49 (231)	-	$65 (305)	$40 (183)	0.275
Total healthcare cost per patient, mean (SD) †	$89 (261)	-	$108 (338)	$80 (213)	0.268
Sertraline, n (%)	567 (11.2)	0	192 (11.4)	375 (22.3)	**<0.001** ^**1, 2, 3**^
Paroxetine, n (%)	104 (2.0)	0	30 (1.8)	74 (4.4)	**<0.001** ^**1, 2, 3**^
**Off-Label Medications, n (%)**	**2460 (48.5)**	**531 (31.0)**	**650 (38.7)**	**1279 (76.1)**	**<0.001** ^**1, 2, 3**^
Number of medications per patient, mean (SD) ^#^	6.3 (11.6)	2.4 (5.9)	4.0 (8.7)	12.6 (15.3)	**<0.001** ^**1, 2, 3**^
Number of medications per patient, mean (SD) †	13.0 (13.8)	7.6 (8.6)	10.4 (11.4)	16.6 (15.5)	**<0.001** ^**1, 2, 3**^
Cost per patient from payer perspective, mean (SD) †	$343 (2016)	$157 (668)	$226 (1388)	$480 (2573)	**≤0.015** ^**2, 3**^
Total healthcare cost per patient, mean (SD) †	$460 (2085)	$227 (718)	$319 (1460)	$628 (2648)	**≤0.003** ^**2, 3**^
Bupropion, n (%)	654 (12.9)	96 (5.6)	130 (7.7)	428 (25.5)	**≤0.013** ^**1, 2, 3**^
Escitalopram, n (%)	610 (12.0)	134 (7.8)	160 (9.5)	316 (18.8)	**<0.001** ^**2, 3**^
Fluoxetine, n (%)	456 (9.0)	77 (4.5)	108 (6.4)	271 (16.1)	**≤0.013** ^**1, 2, 3**^
Buspirone, n (%)	412 (8.1)	71 (4.1)	102 (6.1)	239 (14.2)	**≤0.011** ^**1, 2, 3**^
Prazosin, n (%)	338 (6.7)	32 (1.9)	73 (4.3)	233 (13.9)	**<0.001** ^**2, 3**^
**Any Medication Treatment (FDA-approved or Off-label), n (%)**	**2689 (53.0)**	**531 (31.0)**	**765 (45.5)**	**1393 (82.9)**	**<0.001** ^**1, 2, 3**^
Number of medications per patient, mean (SD) ^#^	7.3 (12.4)	2.4 (5.9)	5.0 (9.5)	14.6 (15.8)	**<0.001** ^**1, 2, 3**^
Number of medications per patient, mean (SD) †	13.8 (14.1)	7.6 (8.6)	11.1 (11.5)	17.6 (15.8)	**<0.001** ^**1, 2, 3**^
Cost per patient from payer perspective, mean (SD) †	$326 (1934)	$157 (668)	$210 (1291)	$453 (2470)	**≤0.008** ^**2, 3**^
Total healthcare cost per patient, mean (SD) †	$442 (2001)	$227 (718)	$301 (1361)	$602 (2543)	**≤0.001** ^**2, 3**^
**Psychotherapy, n (%)**	**2678 (52.8)**	**0**	**1258 (74.8)**	**1420 (84.5)**	**<0.001** ^**1, 2, 3**^
Number of psychotherapy sessions per patient, mean (SD) ^#^	11.3 (21.2)	0	14.0 (21.6)	20.1 (26.1)	**<0.001** ^**1, 2, 3**^
Number of psychotherapy sessions per patient, mean (SD) †	21.4 (25.3)	-	18.7 (23.2)	23.7 (26.8)	**<0.001** ^**3**^
Time-to-psychotherapy (days), mean (SD) †	42.6 (113.9)	-	33.1 (102.3)	50.9 (122.6)	**<0.001** ^**3**^
Cost per patient from payer perspective, mean (SD) †	$1950 (3736)	-	$1545 (3043)	$2309 (4226)	**<0.001** ^**3**^
Total healthcare cost per patient, mean (SD) †	$2501 (4152)	-	$2077 (3478)	$2877 (4638)	**<0.001** ^**3**^
Days between two psychotherapy sessions, mean (SD) •	18.5 (39.4)	-	18.0 (38.4)	19.1 (40.6)	**0.005** ^**3**^
Individual psychotherapy, n (%)	2660 (52.4)	0	1249 (74.3)	1411 (83.9)	**<0.001** ^**1, 2, 3**^
Group psychotherapy, n (%)	187 (3.7)	0	41 (2.4)	146 (8.7)	**<0.001** ^**1, 2, 3**^
Family psychotherapy, n (%)	183 (3.6)	0	86 (5.1)	97 (5.8)	**<0.001** ^**1, 2**^
Interactive complexity psychiatry services, n (%)	128 (2.5)	0	43 (2.6)	85 (5.1)	**<0.001** ^**1, 2, 3**^
Pharmacological management during psychotherapy, n (%)	2 (0.0)	0	1 (0.1)	1 (0.1)	0.600
**Combination of Any Medication and Psychotherapy Treatments, n (%)**	**1725 (34.0)**	**0**	**535 (31.8)**	**1190 (70.8)**	**<0.001** ^**1, 2, 3**^
Combination of FDA-approved medications and psychotherapy, n (%)	538 (10.6)	0	161 (9.6)	377 (22.4)	**<0.001** ^**1, 2, 3**^
Combination of off-label medications and psychotherapy, n (%)	1548 (30.5)	0	454 (27.0)	1094 (65.1)	**<0.001** ^**1, 2, 3**^
**Any Treatment (Medication or Psychotherapy), n (%)**	**3642 (71.7)**	**531 (31.0)**	**1488 (88.5)**	**1623 (96.5)**	**<0.001** ^**1, 2, 3**^
FDA-approved treatments (FDA-approved medications or psychotherapy), n (%)	2796 (55.1)	0	1315 (78.2)	1481 (88.1)	**<0.001** ^**1, 2, 3**^

*Chi-square test was performed for categorical variables. ANOVA with Tamhane’s T2 post hoc test (three-cohort comparison) and independent sample t-test (two-cohort comparison) were performed for continuous variables.

^#^ Across the total sample

^†^ Across the sub-sample of patients who had at least one claim for respective treatment category (FDA-approved, off-label, or psychotherapy)

• Across the population of patients who had at least two psychotherapy sessions during the follow-up

^1^ Baseline PTSD vs. PTSD without Comorbidities, p<0.05

^2^ Baseline PTSD vs. PTSD with Comorbidities, p<0.05

^3^ PTSD without Comorbidities vs. PTSD with Comorbidities, p<0.05

In general, low adherence to pharmacological treatment was noted within the study sample, with PDC values ranging from 0.25 for prazosin to 0.40 for sertraline during the 24-month follow-up. The proportion of adherent patients (PDC ≥0.80) in the total sample ranged between 8.0% (prazosin and buspirone) and 17.5% (escitalopram). Although PTSD patients with severe mental health comorbidities numerically had the lowest adherence estimates for most medications (except for prazosin and bupropion), between-cohort statistical significance was not reached. The exception was escitalopram with a significantly lower rate of adherent patients in the PwC (13.9%) than in the PwoC cohort (23.8%, p = 0.007). All medication adherence measures were estimated among patients with at least one respective prescription claim during the 24-month follow-up; see Table 4.

**Table 4:** Treatment adherence during the 24-month post-index period among the matched population.

	Total Sample (N = 5076)	Baseline PTSD (N = 1714)	PTSD without Comorbidities (N = 1681)	PTSD with Comorbidities (N = 1681)	P-value*
**FDA-Approved Medications**					
≥1 **sertraline** prescription, n (%) ^#^	567 (11.2)	0	192 (11.4)	375 (22.3)	**<0.001** ^**1, 2**^^,^ ^**3**^
PDC, mean (SD) †	0.40 (0.32)	-	0.41 (0.32)	0.39 (0.31)	0.519
Patients with PDC ≥0.80, n (%) †	97 (17.1)	-	36 (18.8)	61 (16.3)	0.457
≥1 **paroxetine** prescription, n (%) ^#^	104 (2.0)	0	30 (1.8)	74 (4.4)	**<0.001** ^**1, 2**^^,^ ^**3**^
PDC, mean (SD) †	0.37 (0.31)	-	0.45 (0.31)	0.33 (0.30)	0.083
Patients with PDC ≥0.80, n (%) †	15 (14.4)	-	5 (16.7)	10 (13.5)	0.678
**Off-Label Medications (Top 5 utilized medications during the 24-month follow-up period)**					
≥1 **bupropion** prescription, n (%) ^#^	654 (12.9)	96 (5.6)	130 (7.7)	428 (25.5)	**≤0.013** ^**1, 2**^^,^ ^**3**^
PDC, mean (SD) †	0.39 (0.31)	0.34 (0.31)	0.38 (0.31)	0.41 (0.31)	0.154
Patients with PDC ≥0.80, n (%) †	103 (15.7)	11 (11.5)	22 (16.9)	70 (16.4)	0.453
≥1 **escitalopram** prescription, n (%) ^#^	610 (12.0)	134 (7.8)	160 (9.5)	316 (18.8)	**<0.001** ^**2**^^,^ ^**3**^
PDC, mean (SD) †	0.37 (0.32)	0.38 (0.33)	0.39 (0.34)	0.36 (0.30)	0.719
Patients with PDC ≥0.80, n (%) †	107 (17.5)	25 (18.7)	38 (23.8)	44 (13.9)	**0.007** ^**3**^
≥1 **fluoxetine** prescription, n (%) ^#^	456 (9.0)	77 (4.5)	108 (6.4)	271 (16.1)	**≤0.013** ^**1, 2**^^,^ ^**3**^
PDC, mean (SD) †	0.37 (0.32)	0.39 (0.33)	0.40 (0.34)	0.36 (0.32)	0.754
Patients with PDC ≥0.80, n (%) †	73 (16.0)	13 (16.9)	21 (19.4)	39 (14.4)	0.468
≥1 **buspirone** prescription, n (%) ^#^	412 (8.1)	71 (4.1)	102 (6.1)	239 (14.2)	**≤0.011** ^**1, 2**^^,^ ^**3**^
PDC, mean (SD) †	0.27 (0.27)	0.28 (0.29)	0.27 (0.30)	0.27 (0.26)	0.997
Patients with PDC ≥0.80, n (%) †	33 (8.0)	6 (8.5)	10 (9.8)	17 (7.1)	0.696
≥1 **prazosin** prescription, n (%) ^#^	338 (6.7)	32 (1.9)	73 (4.3)	233 (13.9)	**<0.001** ^**2**^^,^ ^**3**^
PDC, mean (SD) †	0.25 (0.26)	0.16 (0.24)	0.20 (0.21)	0.27 (0.27)	0.060
Patients with PDC ≥0.80, n (%) †	27 (8.0)	2 (6.3)	2 (2.7)	23 (9.9)	0.136

*Chi-square test was performed for categorical variables. ANOVA with Tamhane’s T2 post hoc test (three-cohort comparison) and independent sample t-test (two-cohort comparison) were performed for continuous variables.

^#^ Across the total sample

^†^ Across the sub-sample of patients who had at least one claim for respective treatment category (FDA-approved or off-label medications)

^1^ Baseline PTSD vs. PTSD without Comorbidities, p<0.05

^2^ Baseline PTSD vs. PTSD with Comorbidities, p<0.05

^3^ PTSD without Comorbidities vs. PTSD with Comorbidities, p<0.05

### SUD/AUD sub-sample

Out of 31326 patients in the final sample of non-matched patients, 3776 patients (85 patients in BP, 537 in PwoC, and 3154 in PwC cohort) were identified with a positive history of SUD/AUD in the 12-month pre-index period or were diagnosed with SUD/AUD during the 24-month post-index period.

Demographic characteristics of SUD/AUD patients are presented in S11 Table in S1 File. Patients with PTSD and SUD/AUD were 35.2 years of age, with 59.5% being women. Patients in the PwC cohort (34.6 years) were significantly younger than patients in the BP and PwoC cohorts (38.7 and 38.3 years, respectively; p≤0.024). The lowest proportion of men with PTSD and SUD/AUD diagnosis was observed in the PwC cohort (36.7%) compared to both BP and PwoC cohorts (67.1% and 58.5%, respectively; both p<0.001). A higher rate of patients with severe mental health comorbidities resided in the Northeast region (15.2%) than those without comorbidities (11.7%, p = 0.038). No other statistically significant differences were reported for patients’ residence and type of healthcare coverage between study cohorts.

The highest proportion of all patients was observed in the CCI = 0 category (71.1%). There were no differences in CCI scores between the study cohorts, except for CCI category 4+ with a significantly higher rate of patients in the BP cohort (8.2%) compared to the PwoC (3.4%, p≤0.038) and PwC cohorts (3.8%, p≤0.038). The most reported CCI comorbidities in the total PTSD sample were reported for chronic pulmonary disease (12.2%), mild liver disease (6.4%), and diabetes without chronic complications (5.9%). The rate of chronic pulmonary disease was significantly higher in PwC (12.7%) than in PwoC (9.3%, p = 0.027). The full list of clinical characteristics of patients diagnosed with PTSD and SUD/AUD is presented in S12 Table in S1 File.

PTSD medications were prescribed to 83.6% of the total sample of PTSD patients with SUD/AUD during the 24-month follow-up, with a mean number of 16.2 prescriptions per patient. The utilization rate and prescription count were the highest among PwC (89.4% of patients with a mean of 18.1 prescriptions) compared to the PwoC (57.0% of patients with a mean of 7.2 prescriptions, both p<0.001) and BP cohorts (35.3% of patients with a mean of 3.0 prescriptions, both p<0.001). Healthcare costs of PTSD pharmacological treatments per treated patient were $410 from the payer perspective and $542 in total costs (payer and out-of-pocket).

FDA-approved PTSD medications were used by 26.0% of the sub-analysis sample (mean 1.8 prescriptions per patient). PwC cases were more commonly treated with FDA-approved PTSD medications (28.2%) and had a higher mean number of respective prescriptions (2.0 prescriptions per patient) than the PwoC cohort (16.6% and 0.9 prescriptions per patient, both p<0.001). The most frequently used medication in this category was sertraline (22.5% of sub-analysis sample) with a higher utilization rate in PwC (24.4%) than PwoC patients (15.3%, p<0.001).

Mean FDA-approved medication-related healthcare costs in treated sub-analysis patients were $41 from the payer perspective and $72 in total costs (payer and out-of-pocket). Off-label PTSD medications were prescribed to 78.9% of the sub-analysis sample with a mean number of 14.4 prescriptions per patient. PwC patients more frequently utilized off-label medications (85.0% of patients) with higher mean prescription counts (16.1 prescriptions) than the PwoC and BP cohorts (49.7% and 35.3%, and 6.3 and 3.0 prescriptions, respectively; all p≤0.013). The most prescribed off-label PTSD medication in the sub-analysis sample was bupropion (23.9% of total patients), with significantly more prescriptions in the PwC cohort (27.1% of patients) compared to the PwoC (8.8% of patients, p<0.001) and BP cohorts (3.5% of patients, p<0.001). Healthcare costs associated with off-label medications per treated patient were $421 from the payer perspective and $551 for total costs (payer and out-of-pocket).

Psychotherapy was provided to 82.9% of sub-analysis patients with a mean of 22.0 sessions per patient. The mean time between the index date and first psychotherapy session was 63.6 days in treated patients, while the mean time between two consecutive sessions was 16.2 days in patients with at least 2 psychotherapy claims. A higher rate of PwC patients was treated with psychotherapy (87.1% of cohort) and had a higher mean number of sessions (24.4 sessions per patient) compared to the PwoC cohort (71.5% of cohort with 11.8 sessions per patient, both p<0.001). The time between the index date and first session among treated patients was longer in patients with severe mental health comorbidities than patients without comorbidities (65.4 vs. 50.4 days, respectively; p = 0.029). However, treated PwC patients utilized psychotherapy more often (mean of 16.0 days between two sessions) than PwoC patients (mean of 18.8 days between two sessions, p<0.001). The most common type was individual psychotherapy (81.6% of sub-analysis sample) following the same utilization trend (85.7% of PwC and 70.4% of PwoC, p<0.001).

Payer healthcare costs for sub-analysis patients treated with psychotherapy were $2883, while total healthcare costs were $3490 per patient. Psychotherapy costs stratified by session duration and type are presented in detail in S13 Table in S1 File. Regarding between-cohort differences, there were significantly higher costs of psychotherapy observed in PwC patients ($3089 payer and $3717 total costs) compared to PwoC patients ($1409 payer and $1863 total costs; both p<0.001).

Almost all sub-analysis patients received PTSD treatment (psychotherapy or PTSD medications) during the 24-month follow-up period (94.9% of patients). The highest rate was observed in PwC patients (97.5%) compared to PwoC and BP patients (89.4% and 35.3% respectively, p<0.001). Combination treatment with psychotherapy and PTSD medication was utilized by 71.6% of sub-analysis patients. A combination of PTSD medication and psychotherapy was more commonly provided to PwC patients (79.0% of patients) than PwoC patients (39.1% of patients, p<0.001). All PTSD treatment characteristics over the 24-month follow-up among patients with PTSD and SUD/AUD diagnosis are shown in Table 5.

**Table 5:** Treatment characteristics of patients with PTSD and SUD/AUD diagnosis during the 24-month post-index period.

	Total Sample (N = 3776)	Baseline PTSD (N = 85)	PTSD without Comorbidities (N = 537)	PTSD with Comorbidities (N = 3154)	P-value*
**FDA-Approved Medications, n (%)**	**980 (26.0)**	**0**	**89 (16.6)**	**891 (28.2)**	**<0.001** ^**1**^^,^ ^**2**^^,^ ^**3**^
Number of medications per patient, mean (SD) ^#^	1.8 (4.5)	0	0.9 (3.5)	2.0 (4.7)	**<0.001** ^**1**^^,^ ^**2**^^,^ ^**3**^
Number of medications per patient, mean (SD) †	6.8 (6.6)	-	5.7 (6.8)	6.9 (6.5)	0.083
Cost per patient from payer perspective, mean (SD) †	$41 (168)	-	$33 (91)	$42 (174)	0.625
Total healthcare cost per patient, mean (SD) †	$72 (195)	-	$59 (107)	$73 (202)	0.510
Sertraline, n (%)	851 (22.5)	0	82 (15.3)	769 (24.4)	**<0.001** ^**1**^^,^ ^**2**^^,^ ^**3**^
Paroxetine, n (%)	155 (4.1)	0	9 (1.7)	146 (4.6)	**≤0.032** ^**2**^^,^ ^**3**^
**Off-Label Medications, n (%)**	**2979 (78.9)**	**30 (35.3)**	**267 (49.7)**	**2682 (85.0)**	**≤0.013** ^**1**^^,^ ^**2**^^,^ ^**3**^
Number of medications per patient, mean (SD) ^#^	14.4 (16.9)	3.0 (7.2)	6.3 (11.9)	16.1 (17.2)	**≤0.002** ^**1**^^,^ ^**2**^^,^ ^**3**^
Number of medications per patient, mean (SD) †	18.3 (17.0)	8.5 (10.1)	12.6 (14.3)	19.0 (17.2)	**<0.001** ^**2**^^,^ ^**3**^
Cost per patient from payer perspective, mean (SD) †	$421 (1698)	$210 (956)	$388 (2503)	$427 (1603)	0.545
Total healthcare cost per patient, mean (SD) †	$551 (1876)	$277 (994)	$509 (3101)	$558 (1715)	0.359
Bupropion, n (%)	904 (23.9)	3 (3.5)	47 (8.8)	854 (27.1)	**<0.001** ^**2**^^,^ ^**3**^
Prazosin, n (%)	740 (19.6)	1 (1.2)	58 (10.8)	681 (21.6)	**≤0.002** ^**1, 2, 3**^
Escitalopram, n (%)	728 (19.3)	7 (8.2)	63 (11.7)	658 (20.9)	**≤0.004** ^**2**^^,^ ^**3**^
Buspirone, n (%)	706 (18.7)	5 (5.9)	48 (8.9)	653 (20.7)	**<0.001** ^**2**^^,^ ^**3**^
Fluoxetine, n (%)	628 (16.6)	4 (4.7)	46 (8.6)	578 (18.3)	**<0.001** ^**2**^^,^ ^**3**^
**Any Medication Treatment (FDA-Approved or Off-Label), n (%)**	**3156 (83.6)**	**30 (35.3)**	**306 (57.0)**	**2820 (89.4)**	**<0.001** ^**1**^^,^ ^**2**^^,^ ^**3**^
Number of medications per patient, mean (SD) ^#^	16.2 (17.5)	3.0 (7.2)	7.2 (12.3)	18.1 (17.8)	**<0.001** ^**1**^^,^ ^**2**^^,^ ^**3**^
Number of medications per patient, mean (SD) †	19.4 (17.5)	8.5 (10.1)	12.7 (14.0)	20.2 (17.7)	**<0.001** ^**2**^^,^ ^**3**^
Cost per patient from payer perspective, mean (SD) †	$410 (1656)	$210 (956)	$348 (2342)	$419 (1570)	0.573
Total healthcare cost per patient, mean (SD) †	$542 (1830)	$277 (994)	$461 (2901)	$554 (1681)	0.371
**Psychotherapy, n (%)**	**3131 (82.9)**	**0**	**384 (71.5)**	**2747 (87.1)**	**<0.001** ^**1**^^,^ ^**2**^^,^ ^**3**^
Number of psychotherapy sessions per patient, mean (SD) ^#^	22.0 (30.2)	0	11.8 (19.4)	24.4 (31.5)	**<0.001** ^**1**^^,^ ^**2**^^,^ ^**3**^
Number of psychotherapy sessions per patient, mean (SD) †	26.6 (31.3)	-	16.5 (21.2)	28.0 (32.2)	**<0.001**
Time-to-psychotherapy (days), mean (SD) †	63.6 (135.6)	-	50.4 (123.4)	65.4 (137.1)	**0.029**
Cost per patient from payer perspective, mean (SD) †	$2883 (5587)	-	$1409 (2476)	$3089 (5863)	**<0.001** ^**3**^
Total healthcare cost per patient, mean (SD) †	$3490 (6094)	-	$1863 (2959)	$3717 (6379)	**<0.001** ^**3**^
Days between two psychotherapy sessions, mean (SD) •	16.2 (39.1)	-	18.8 (43.3)	16.0 (38.7)	**<0.001** ^**3**^
Individual psychotherapy, n (%)	3081 (81.6)	0	378 (70.4)	2703 (85.7)	**<0.001** ^**1**^^,^ ^**2**^^,^ ^**3**^
Group psychotherapy, n (%)	691 (18.3)	0	47 (8.8)	644 (20.4)	**≤0.001** ^**1**^^,^ ^**2**^^,^ ^**3**^
Family psychotherapy, n (%)	286 (7.6)	0	28 (5.2)	258 (8.2)	**≤0.023** ^**1**^^,^ ^**2**^^,^ ^**3**^
Interactive complexity psychiatry services, n (%)	183 (4.8)	0	11 (2.0)	172 (5.5)	**≤0.023** ^**2**^^,^ ^**3**^
Pharmacological management during psychotherapy, n (%)	13 (0.3)	0	0	13 (0.4)	0.276
**Combination of any Medication and Psychotherapy Treatments, n (%)**	**2702 (71.6)**	**0**	**210 (39.1)**	**2492 (79.0)**	**<0.001** ^**1**^^,^ ^**2**^^,^ ^**3**^
Combination of FDA-approved medications and psychotherapy, n (%)	841 (22.3)	0	54 (10.1)	787 (25.0)	**≤0.001** ^**1**^^,^ ^**2**^^,^ ^**3**^
Combination of off-label medications and psychotherapy, n (%)	2560 (67.8)	0	186 (34.6)	2374 (75.3)	**<0.001** ^**1**^^,^ ^**2**^^,^ ^**3**^
**Any Treatment (Medication or Psychotherapy), n (%)**	**3585 (94.9)**	**30 (35.3)**	**480 (89.4)**	**3075 (97.5)**	**<0.001** ^**1**^^,^ ^**2**^^,^ ^**3**^
FDA-approved treatments (FDA-approved medications or psychotherapy), n (%)	3270 (86.6)	0	419 (78.0)	2851 (90.4)	**<0.001** ^**1**^^,^ ^**2**^^,^ ^**3**^

*Chi-square test was performed for categorical variables. ANOVA with Tamhane’s T2 post hoc test (three-cohort comparison) and independent sample t-test (two-cohort comparison) were performed for continuous variables.

^#^ Across the total sample

^†^ Across the sub-sample of patients who had at least one claim for respective treatment category (FDA-approved, off-label, or psychotherapy)

• Across the population of patients who had at least two psychotherapy sessions during the follow-up

^1^ Baseline PTSD vs. PTSD without Comorbidities, p<0.05

^2^ Baseline PTSD vs. PTSD with Comorbidities, p<0.05

^3^ PTSD without Comorbidities vs. PTSD with Comorbidities, p<0.05

In general, low adherence to pharmacological treatments was also demonstrated in the sub-analysis sample with mean PDC values ranging between 0.23 for prazosin to 0.34 for bupropion and escitalopram. The rates of adherent patients (PDC ≥0.80) were also low and varied from 4.7% for prazosin to 11.4% for escitalopram. There were no statistically significant differences in results between study cohorts for FDA-approved medications. However, the PwC cohort had numerically lower rates of adherent patients on sertraline (9.5% compared to 14.6% in PwoC) and paroxetine (6.2% compared to 11.1% in PwoC). Regarding off-label medications, mean PDC value for prazosin was significantly higher in PwC patients (0.23) than in PwoC patients (0.16, p = 0.013). On the other hand, of patients treated with escitalopram, the PwC cohort had significantly lower adherence rates (10.2%) compared to the PwoC cohort (25.4%, p<0.001). Medication adherence estimates per medication were explored only among patients with at least one respective prescription claim during the 24-month post-index period (Table 6).

**Table 6:** Treatment adherence during the 24-month post-index period among patients with PTSD and SUD/AUD diagnosis.

	Total Sample (N = 3776)	Baseline PTSD (N = 85)	PTSD without Comorbidities (N = 537)	PTSD with Comorbidities (N = 3154)	P-value*
**FDA-Approved Medications**					
≥1 **sertraline** prescription, n (%) ^#^	851 (22.5)	0	82 (15.3)	769 (24.4)	**<0.001** ^**1**^^,^ ^**2**^^,^ ^**3**^
PDC, mean (SD) †	0.32 (0.28)	-	0.29 (0.29)	0.32 (0.28)	0.341
Patients with PDC ≥0.80, n (%) †	85 (10.0)	-	12 (14.6)	73 (9.5)	0.140
≥1 **paroxetine** prescription, n (%) ^#^	155 (4.1)	0	9 (1.7)	146 (4.6)	**≤0.032** ^**2**^^,^ ^**3**^
PDC, mean (SD) †	0.29 (0.27)	-	0.30 (0.32)	0.29 (0.26)	0.928
Patients with PDC ≥0.80, n (%) †	10 (6.5)	-	1 (11.1)	9 (6.2)	0.558
**Off-Label Medications (Top 5 utilized medications during the 24-month follow-up period)**					
≥1 **bupropion** prescription, n (%) ^#^	904 (23.9)	3 (3.5)	47 (8.8)	854 (27.1)	**<0.001** ^**2**^^,^ ^**3**^
PDC, mean (SD) †	0.34 (0.29)	0.16 (0.07)	0.28 (0.27)	0.34 (0.29)	0.110
Patients with PDC ≥0.80, n (%) †	101 (11.2)	0	4 (8.5)	97 (11.4)	0.690
≥1 **prazosin** prescription, n (%) ^#^	740 (19.6)	1 (1.2)	58 (10.8)	681 (21.6)	**≤0.002** ^**1**^^,^ ^**2**^^,^ ^**3**^
PDC, mean (SD) †	0.23 (0.24)	0.04 (-)	0.16 (0.21)	0.23 (0.24)	**0.013** ^**3**^
Patients with PDC ≥0.80, n (%) †	35 (4.7)	0	1 (1.7)	34 (5.0)	0.518
≥1 **escitalopram** prescription, n (%) ^#^	728 (19.3)	7 (8.2)	63 (11.7)	658 (20.9)	**≤0.004**^**2**^^,^ ^**3**^
PDC, mean (SD) †	0.34 (0.29)	0.28 (0.29)	0.41 (0.34)	0.34 (0.28)	0.329
Patients with PDC ≥0.80, n (%) †	83 (11.4)	0	16 (25.4)	67 (10.2)	**<0.001** ^**3**^
≥1 **buspirone** prescription, n (%) ^#^	706 (18.7)	5 (5.9)	48 (8.9)	653 (20.7)	**<0.001** ^**2**^^,^ ^**3**^
PDC, mean (SD) †	0.24 (0.25)	0.34 (0.31)	0.25 (0.26)	0.24 (0.24)	0.876
Patients with PDC ≥0.80, n (%) †	35 (5.0)	1 (20.0)	2 (4.2)	32 (4.9)	0.291
≥1 **fluoxetine** prescription, n (%) ^#^	628 (16.6)	4 (4.7)	46 (8.6)	578 (18.3)	**<0.001** ^**2**^^,^ ^**3**^
PDC, mean (SD) †	0.33 (0.28)	0.38 (0.37)	0.32 (0.31)	0.33 (0.28)	0.992
Patients with PDC ≥0.80, n (%) †	60 (9.6)	1 (25.0)	7 (15.2)	52 (9.0)	0.221

*Chi-square test was performed for categorical variables. ANOVA with Tamhane’s T2 post hoc test (three-cohort comparison) and independent sample t-test (two-cohort comparison) were performed for continuous variables.

^#^ Across the total sample

^†^ Across the sub-sample of patients who had at least one claim for respective treatment category (FDA-approved or off-label medication)

^1^ Baseline PTSD vs. PTSD without Comorbidities, p<0.05

^2^ Baseline PTSD vs. PTSD with Comorbidities, p<0.05

^3^ PTSD without Comorbidities vs. PTSD with Comorbidities, p<0.05

## Discussion

To the best of our knowledge, this is the first study that evaluated treatment characteristics and adherence measures among US patients diagnosed with chronic PTSD stratified by disease severity. In addition, study outcomes were evaluated among a sub-group of patients with PTSD and SUD/AUD.

The primary analysis in this study demonstrated that 71.7% of patients had PTSD treatment of some type during the 24-month follow-up period. A small proportion of patients were treated with FDA-approved medications (12.9%), and a considerably higher proportion of patients were treated with off-label medications (48.5%). Adherence to pharmacological treatments was suboptimal in relation to a target of 0.80 (mean PDC 0.25–0.40). Treated patients covered with medication more than 80.0% of the time during the 24-month follow-up period ranged from 8.0–17.5%. The proportion of adherent patients to escitalopram (PDC ≥0.80) was significantly lower in PwC compared to PwoC (13.9% vs. 23.8%). Patients with severe mental health comorbidities were also less adherent to most other observed medications; however, the statistical significance threshold was not reached.

Across all cohorts, 52.8% of patients received psychotherapy, with psychotherapy being the most utilized PTSD treatment option. Psychotherapy sessions were more commonly utilized by PwC than PwoC patients (20.1 sessions vs. 14.0 sessions), while the time-to-first psychotherapy session was longer in cases with severe mental health comorbidities compared to those without comorbidities (mean 65.4 days vs. 50.4 days). The mean 24-month payer-related healthcare costs per patient treated with FDA-approved medications, off-label medications, and psychotherapy were $49, $343, and $1950 respectively. Including patients’ out-of-pocket costs, the corresponding 24-month total healthcare costs per treated patient were $89 with FDA-approved medications, $460 with off-label medications, and $2501 with psychotherapy.

In the sub-analysis, only 5.1% of patients with PTSD and an SUD/AUD diagnosis did not have any treatment during the 24-month follow-up period. On the other hand, patients with PTSD and SUD/AUD were mostly treated with psychotherapy (82.9%) followed by off-label medications (78.9%), while the lowest proportion of patients were treated with FDA-approved medications (26.0%). As in the primary analysis, the highest rate of patients treated for PTSD was observed among the PwC cohort (97.5%). It was estimated that 71.6% of patients with PTSD and SUD/AUD had concomitant treatment consisting of PTSD medications and psychotherapy.

Similar to the general PTSD population, adherence to pharmacological treatments was low, with mean PDC scores ranging from 0.23 to 0.34, and the proportion of adherent patients ranging from 4.7% to 11.4%. Slightly higher total healthcare costs were reported in SUD/AUD patients than in the general PTSD population, with per patient costs of $72 for FDA-approved medications, $551 for off-label medications, and $3490 for psychotherapy sessions during the 24-month follow-up period.

VA/DoD guidelines from 2023 recommend manualized trauma-focused psychotherapy as first-line treatment for PTSD, with individual psychotherapy recommended over pharmacological treatments. The guidelines are based on evidence that trauma-focused psychotherapy imparts greater change on core PTSD symptoms than pharmacotherapies and that these improvements persist for longer periods. More specifically, individual cognitive processing therapy (CPT), eye movement desensitization and reprocessing (EMDR), prolonged exposure (PE), Ehler’s cognitive therapy, present-centered therapy, and written exposure therapy were established as effective psychotherapy in PTSD management [8]. APA guidelines from 2017 also recommend psychotherapy over pharmacological management for the treatment of PTSD. APA guidelines provide strong recommendations for cognitive behavioral therapy (CBT), CPT, cognitive therapy, and PE, while brief eclectic psychotherapy, EMDR therapy, and narrative exposure therapy gained conditional recommendations [7]. A systematic literature review (SLR) and meta-analysis conducted by Cusack et al. provided high strength of evidence for the efficacy of manualized version PE, cognitive therapy, and CPT, and low-to-moderate strength of evidence for the efficacy of CBT-mixed therapies, EMDR and narrative exposure therapy in the management of PTSD [19]. Another SLR with meta-analysis conducted by Watts et al. demonstrated that cognitive therapy, exposure therapy, and EMDR were effective psychotherapy types for PTSD [20]. Our study findings showed that individual psychotherapy sessions were the most utilized PTSD treatment among patients with PTSD (52.8%), as well as in the sub-sample of patients with PTSD and diagnosed SUD/AUD (82.9%), which is in line with guideline recommendations and previous study results.

Paroxetine and sertraline are the only medications approved by the FDA for the treatment of PTSD in the US. The most recent VA/DoD guideline provides strong recommendation for these medications in PTSD management [8]. A meta-analysis conducted by Watts et al. showed that paroxetine and sertraline were effective pharmacological treatment options for PTSD [20]. This retrospective study conducted using real-world data reported that only 13.0% of the PwoC cohort and 26.1% of the PwC cohort were treated with these medications.

Many pharmacological treatments are not indicated for PTSD but are used in clinical practice to treat PTSD symptoms. VA/DoD guidelines do not provide recommendations for or against the following off-label medications for the treatment of PTSD due to insufficient evidence: amitriptyline, bupropion, buspirone, citalopram, desvenlafaxine, duloxetine, escitalopram, eszopiclone, fluoxetine, imipramine, mirtazapine, lamotrigine, nefazodone, olanzapine, phenelzine, pregabalin, rivastigmine, topiramate, and quetiapine. It was mentioned that different concerns exist for each medication, including the level of evidence, side effect profiles, and potential benefits for alternative uses [8]. Our study demonstrated that almost half of patients with PTSD in the general sample (48.5%) utilized some of the listed off-label medications during the 24-month period after established PTSD diagnosis, while even more were treated with off-label medications in the sub-group of patients with PTSD and SUD/AUD (78.9%). Simpson et al. reported that 84.0% of individuals with co-occurring PTSD and SUD received at least one mental health treatment [21], which is in line with the findings of our sub-analysis that yielded 94.9% of patients treated with PTSD medications or psychotherapy.

Several studies evaluated adherence to medication treatment in patients with PTSD. Our study findings suggested very low adherence to prescribed medications (both FDA-approved and off-label) among patients with PTSD, which aligns with publicly available literature. Kronish et al. reported that patients with PTSD were associated with medication non-adherence independent of psychiatric and medical comorbidities that significantly impacted treatment efficacy and disease morbidity. It was noted that 12.0% of patients did not take their medications as prescribed, and 41.0% of patients reported forgetting to take their medications [22]. In addition, Salas et al. reported low adherence (defined as PDC ≥0.80) to antidepressant medications in a PTSD sample with or without significantly meaningful change in PTSD symptoms (53.5% and 39.3%, respectively) [23]. Rakofsky et al. emphasized the high risk of treatment non-adherence among patients with PTSD and comorbid bipolar disorder due to several factors, such as early childhood trauma, and drug or clinician characteristics [24].

This real-world evidence study provides a significant addendum to the current literature, with a comprehensive overview of the treatment patterns, adherence measures, and associated healthcare costs (total and payer-related) among patients with PTSD, and patients with PTSD and SUD/AUD comorbidity. Additionally, study outcomes were explored within different PTSD categories based on disease severity (defined by comorbid severe mental health conditions), showing that PwC significantly differed in terms of utilization rates (psychotherapy and medication) and treatment costs, compared to the BP and PwoC cohorts.

### Limitations

To the best of our knowledge, this is the first real-world evidence analysis of treatment characteristics and adherence measures among US patients diagnosed with PTSD stratified by disease severity. Nonetheless, several study limitations should be mentioned for results interpretation. First is the limitation related to the nature and characteristics of retrospectively gathered real-world data and coding system restrictions. The claims are primarily collected for billing purposes; therefore, data entry errors and miscoding, duplicates, or negative-input claims may have occurred. This was addressed by employing data cleaning, performing a precise patient selection process, and using complex methods, such as propensity-score matching, to minimize selection bias and balance the differences between study cohorts. Second, there is a lack of data related to PTSD severity, and some assumptions have been made to stratify patients. More PwC patients were assumed to have at least one concomitant diagnosis of MDD, schizophrenia, or bipolar disorder during the 24-month period after the first PTSD diagnosis. On the other hand, patients with asymptomatic or non-severe PTSD were cases with a lack of the abovementioned comorbidities (BP and PwoC). Third, off-label medications were selected based on the most recent PTSD treatment guidelines available in the US during the study conduction. There is a possibility that some of the assessed off-label medications were not prescribed particularly for PTSD management due to the lack of a PTSD-related indication. Hence, the authors evaluated only the most relevant off-label medications during the 24-month period after index PTSD diagnosis. Furthermore, PSM analysis was employed to balance differences in demographic and clinical characteristics and to diminish their potential impact on the off-label medication prescribing rates among cohorts. Fourth, there is a lack of coding related to specific psychotherapy modalities. Hence, further evaluation of the psychotherapy modality utilized in PTSD management was not possible. The fifth study limitation is related to the study findings’ generalizability, as the analysis was conducted in a sample of commercially insured patients without the inclusion of VA, Medicare, or Medicaid populations.

## Conclusions

The real-world findings indicate that the majority of assessed patients with PTSD were managed with continuous psychotherapy during the follow-up period. The mean healthcare cost of psychotherapy sessions (regardless of duration) was numerically higher than the mean treatment costs per patient treated with FDA-approved medications, indicating that psychotherapy leads to a significant economic burden among patients with PTSD. PTSD patients with severe mental health comorbidities had significantly higher treatment utilization rates and associated costs than those in the PwoC and BP cohorts. Patients with SUD/AUD and concomitant PTSD demonstrated even greater treatment consumption and respective costs. Low rates of patients treated with FDA-approved medications and high rates of off-label medication utilization, with generally low adherence to all pharmacological treatments in the real-world setting, emphasizes the need for more efficacious treatment.

The findings indicate patients with severe mental comorbidities had a high medication burden, which may lead to augmentation of adverse events and unnecessary costs. On the other hand, the analysis revealed there is a high proportion of PTSD-diagnosed patients without treatment and medical follow-up in the US healthcare system implying undertreatment in some patients. This real-world analysis provides a comprehensive overview of PTSD patient characteristics and treatment pathways, and identified potential areas for improvement relevant to treating patients with PTSD. Novel treatment should focus on mitigating the symptoms of chronic PTSD, improving patients’ quality of life, and rationalizing the resource consumption related to PTSD management, especially for severe PTSD cases.

## Supporting information

S1 File (DOCX)

## References

[1] Merians AN, Spiller T, Harpaz-Rotem I, Krystal JH, Pietrzak RH. Post-traumatic stress disorder. Med Clin North Am. 2023;107(1):85–99. doi: 10.1016/j.mcna.2022.04.003 36402502

[2] Yehuda R, Hoge CW, McFarlane AC, Vermetten E, Lanius RA, Nievergelt CM, et al. Post-traumatic stress disorder. Nat Rev Dis Primers. 2015;1(1):15057. 27189040 10.1038/nrdp.2015.57

[3] Schein J, Houle C, Urganus A, Cloutier M, Patterson-Lomba O, Wang Y, et al. Prevalence of post-traumatic stress disorder in the United States: a systematic literature review. Curr Med Res Opin. 2021;37(12):2151–61. doi: 10.1080/03007995.2021.1978417 34498953

[4] Lehavot K, Katon JG, Chen JA, Fortney JC, Simpson TL. Post-traumatic stress disorder by gender and veteran status. Am J Prev Med. 2018;54(1):e1–e9. doi: 10.1016/j.amepre.2017.09.008 29254558 PMC7217324

[5] Kessler RC, Rose S, Koenen KC, Karam EG, Stang PE, Stein DJ, et al. How well can post-traumatic stress disorder be predicted from pre-trauma risk factors? An exploratory study in the WHO World Mental Health Surveys. World Psychiatry. 2014;13(3):265–74. doi: 10.1002/wps.20150 25273300 PMC4219068

[6] Davis LL, Schein J, Cloutier M, Gagnon-Sanschagrin P, Maitland J, Urganus A, et al. The economic burden of posttraumatic stress disorder in the United States from a societal perspective. J Clin Psychiatry. 2022;83(3). doi: 10.4088/JCP.21m14116 35485933

[7] The American Psychological Association. Clinical Practice Guideline for the Treatment of Posttraumatic Stress Disorder (PTSD) in Adults. 2017. Available from: https://www.apa.org/ptsd-guideline.

[8] US Department of Veteran Affairs. Veteran Affairs/Department of Defense (VA/DoD) clinical practice guideline for management of posttraumatic stress disorder and acute stress disorder. 2023. Available from: https://www.healthquality.va.gov/guidelines/MH/ptsd/VA-DoD-CPG-PTSD-Full-CPGAug242023.pdf.

[9] Bhatt SR, Armstrong M, Parker T, Maviglia M, Kass R, Leeman L, et al. Psychedelic Therapies at the Crossroads of Trauma and Substance Use: Historical Perspectives and Future Directions, Taking a Lead From New Mexico. Front Pharmacol. 2022;13:905753. doi: 10.3389/fphar.2022.905753 35833023 PMC9273054

[10] Smith NDL, Cottler LB. The Epidemiology of Post-Traumatic Stress Disorder and Alcohol Use Disorder. Alcohol Res. 2018;39(2):113–20. 31198651 10.35946/arcr.v39.2.02 PMC6561398

[11] Kessler RC, Sonnega A, Bromet E, Hughes M, Nelson CB. Posttraumatic stress disorder in the National Comorbidity Survey. Arch Gen Psychiatry. 1995;52(12):1048–60. doi: 10.1001/archpsyc.1995.03950240066012 7492257

[12] Cuschieri S. The STROBE guidelines. Saudi J Anaesth. 2019;13(Suppl 1):S31–s4. doi: 10.4103/sja.SJA_543_18 30930717 PMC6398292

[13] Merative. IBM MarketScan Research Databases is now Merative™ MarketScan® Research Databases. 2023. Available from: https://www.merative.com/real-world-evidence.

[14] Cahill SP, Pontoski K. Post-traumatic stress disorder and acute stress disorder I: their nature and assessment considerations. Psychiatry (Edgmont). 2005;2(4):14–25. 21179648 PMC3004735

[15] Seng JS, Clark MK, McCarthy AM, Ronis DL. PTSD and physical comorbidity among women receiving Medicaid: results from service-use data. J Trauma Stress. 2006;19(1):45–56. doi: 10.1002/jts.20097 16568470

[16] Seng JS, Graham-Bermann SA, Clark MK, McCarthy AM, Ronis DL. Posttraumatic stress disorder and physical comorbidity among female children and adolescents: results from service-use data. Pediatrics. 2005;116(6):e767–76. doi: 10.1542/peds.2005-0608 16322133

[17] WebMD Editorial Contributors. Different Types of Health Plans: How They Compare. 2022. Available from: https://www.webmd.com/health-insurance/types-of-health-insurance-plans.

[18] Falkson SR, Srinivasan VN. Health Maintenance Organization. StatPearls. Treasure Island (FL): StatPearls Publishing Copyright © 2024, StatPearls Publishing LLC.; 2024. 32119341

[19] Cusack K, Jonas DE, Forneris CA, Wines C, Sonis J, Middleton JC, et al. Psychological treatments for adults with posttraumatic stress disorder: A systematic review and meta-analysis. Clin Psychol Rev. 2016;43:128–41. doi: 10.1016/j.cpr.2015.10.003 26574151

[20] Watts BV, Schnurr PP, Mayo L, Young-Xu Y, Weeks WB, Friedman MJ. Meta-analysis of the efficacy of treatments for posttraumatic stress disorder. J Clin Psychiatry. 2013;74(6):e541–50. doi: 10.4088/JCP.12r08225 23842024

[21] Simpson TL, Hawrilenko M, Goldberg S, Browne K, Lehavot K, Borowitz M. Treatment receipt patterns among individuals with co-occurring posttraumatic stress disorder (PTSD) and substance use disorders. J Consult Clin Psychol. 2020;88(11):1039–51. doi: 10.1037/ccp0000600 32790452 PMC9851411

[22] Kronish IM, Edmondson D, Li Y, Cohen BE. Post-traumatic stress disorder and medication adherence: Results from the Mind Your Heart Study. J Psychiatr Res. 2012;46(12):1595–9. doi: 10.1016/j.jpsychires.2012.06.011 22809686 PMC3485414

[23] Salas J, Scherrer JF, Tuerk P, van den Berk-Clark C, Chard KM, Schneider FD, et al. Large posttraumatic stress disorder improvement and antidepressant medication adherence. J Affect Disord. 2020;260:119–23. doi: 10.1016/j.jad.2019.08.095 31494363 PMC6803073

[24] Rakofsky JJ, Dunlop BW, Levy ST. Conceptualizing treatment nonadherence in patients with bipolar disorder and PTSD. CNS Spectrums. 2011;16(1):11–20. doi: 10.1017/S1092852912000119 24725297

