# Supplementary material for: Treatment patterns and characteristics of patients with Post-Traumatic Stress Disorder (PTSD): A retrospective claims analysis among commercially insured population
Source: PLoS One. 2024 Oct 30;19(10):e0309704. doi: 10.1371/journal.pone.0309704 (PMC11524461; doi:10.1371/journal.pone.0309704)
Supplement: S1 File — (DOCX) [file pone.0309704.s001.docx]

Supplementary Materials

**Table S1.** ICD-10-CM codes for chronic PTSD

| **ICD-10-CM code** | **Description** |
| --- | --- |
| F43.1 | Posttraumatic stress disorder (PTSD) |
| F43.10 | Posttraumatic stress disorder, unspecified |
| F43.12 | Posttraumatic stress disorder, chronic |

**Table S2.** ICD-10 CM codes for acute PTSD

| **ICD-10-CM code** | **Description** |
| --- | --- |
| F43.11 | Posttraumatic stress disorder, acute |

**Table S3.** ICD-10-CM codes related to cancer diagnosis

| **Diagnosis** | **ICD-10-CM codes** |
| --- | --- |
| Malignancy | C00 C01 C02 C03 C04 C05 C06 C07 C08 C09 C10 C11 C12 C13 C14 C15 C16 C17 C18 C19 C20 C21 C22 C23 C24 C25 C26 C30 C31 C32 C33 C34 C37 C38 C39 C40 C41 C43 C44 C45 C46 C47 C48 C49 C50 C51 C52 C53 C54 C55 C56 C57 C58 C60 C61 C62 C63 C64 C65 C66 C67 C68 C69 C70 C71 C72 C73 C74 C75 C76 C81 C82 C83 C84 C85 C86 C88 C90 C91 C92 C93 C94 C95 C96 C97 D47Z9 C7A00 C7A010 C7A011 C7A012 C7A019 C7A020 C7A021 C7A022 C7A023 C7A024 C7A025 C7A026 C7A029 C7A090 C7A091 C7A092 C7A093 C7A094 C7A095 C7A096 C7A098 C7A1 C7A8 D469 E3121 E3122 E3123 C49A0 C49A1 C49A2 C49A3 C49A4 C49A5 C49A9 C4A0 C4A10 C4A11 C4A111 C4A112 C4A12 C4A121 C4A122 C4A20 C4A21 C4A22 C4A30 C4A31 C4A39 C4A4 C4A51 C4A52 C4A59 C4A60 C4A61 C4A62 C4A70 C4A71 C4A72 C4A8 C4A9 |
| Metastatic solid | C77 C78 C79 C80 C7B00 C7B01 C7B02 C7B03 C7B04 C7B09 C7B1 C7B8 |

**Table S4.** ICD-10CM codes for SUD/AUD

| **Diagnosis** | **ICD-10-CM codes** |
| --- | --- |
| SUD/AUD | F10.1 F10.10 F10.11 F10.12 F10.120 F10.121 F10.129 F10.13 F10.130 F10.131 F10.132 F10.139 F10.14 F10.15 F10.150 F10.151 F10.159 F10.18 F10.180 F10.181 F10.182 F10.188 F10.19 F11.1 F11.10 F11.11 F11.12 F11.120 F11.121 F11.122 F11.129 F11.13 F11.14 F11.15 F11.150 F11.151 F11.159 F11.18 F11.181 F11.182 F11.188 F11.19 F12.1 F12.10 F12.11 F12.12 F12.120 F12.121 F12.122 F12.129 F12.13 F12.15 F12.150 F12.151 F12.159 F12.18 F12.180 F12.188 F12.19 F13.1 F13.10 F13.11 F13.12 F13.120 F13.121 F13.129 F13.13 F13.130 F13.131 F13.132 F13.139 F13.14 F13.15 F13.150 F13.151 F13.159 F13.18 F13.180 F13.181 F13.182 F13.188 F13.19 F14.1 F14.10 F14.11 F14.12 F14.120 F14.121 F14.122 F14.129 F14.13 F14.14 F14.15 F14.150 F14.151 F14.159 F14.18 F14.180 F14.181 F14.182 F14.188 F14.19 F15.1 F15.10 F15.11 F15.12 F15.120 F15.121 F15.122 F15.129 F15.13 F15.14 F15.15 F15.150 F15.151 F15.159 F15.18 F15.180 F15.181 F15.182 F15.188 F15.19 F16.1 F16.10 F16.11 F16.12 F16.120 F16.121 F16.122 F16.129 F16.14 F16.15 F16.150 F16.151 F16.159 F16.18 F16.180 F16.183 F16.188 F16.19 F18.1 F18.10 F18.11 F18.12 F18.120 F18.121 F18.129 F18.14 F18.15 F18.150 F18.151 F18.159 F18.17 F18.18 F18.180 F18.188 F18.19 F19.1 F19.10 F19.11 F19.12 F19.120 F19.121 F19.122 F19.129 F19.13 F19.130 F19.131 F19.132 F19.139 F19.14 F19.15 F19.150 F19.151 F19.159 F19.16 F19.17 F19.18 F19.180 F19.181 F19.182 F19.188 F19.19 |

**Table S5**. ICD-10-CM codes related to MDD, bipolar disorder, and schizophrenia

| **Diagnosis** | **ICD-10-CM codes** |
| --- | --- |
| Bipolar disorder | F3110 F3111 F3112 F3113 F312 F3173 F3174 F3130 F3131 F3132 F314 F315 F3175 F3176 F3160 F3161 F3162 F3163 F3164 F3177 F3178 F319 |
| MDD | F329 F320 F321 F322 F323 F324 F325 F339 F330 F331 F332 F333 F3341 F3342 F341 F329 F32 F320 F321 F322 F323 F324 F325 F33 F330 F331 F332 F333 F334 F3340 F3341 F3342 F338 F339 |
| Schizophrenia | F20 F200 F201 F202 F203 F205 F208 F2081 F2089 F209 F25 F250 F251 F258 F259 |

**Table S6.** NDC codes related to FDA-approved medications for PTSD treatment

| **Medication** | **NDC code** |
| --- | --- |
| Paroxetine | 29320613 29320713 29320813 29321013 29321113 29321120 29321121 29321145 29321159 29321213 29321313 29321325 29321548 29460613 29460713 93711456 93711498 93711556 93711598 93711656 93711698 93712156 93712198 378200305 378200393 378200405 378200493 378200593 378200693 378700110 378700193 378700210 378700293 378700310 378700393 378700410 378700493 403475330 403533718 406200103 406200105 406200190 406209703 406209705 406209790 406209801 406209803 406209805 406209890 406209901 406209903 406209905 406209990 574027930 781196731 781196931 781199101 781199231 904567661 904567761 904567861 904567961 904610961 904611061 904611161 904611261 10544043330 10544080930 12280001615 12280001715 12280001815 13107015405 13107015430 13107015490 13107015505 13107015530 13107015590 13107015599 13107015605 13107015630 13107015690 13107015699 13107015705 13107015730 13107015790 13107015799 16590018115 16590018128 16590018130 16590018160 16590018182 16590018186 16590018190 16590032215 16590032230 16590032256 16590032260 16590032272 16590032290 16590051206 16590051230 16590051260 16590051272 16590051290 16590051330 16590051356 16590051360 16590051372 16590051390 16590051430 16590051460 16590051472 16590051490 16590089630 16714018101 16714018102 16714018103 16714018104 16714018105 16714018201 16714018202 16714018203 16714018204 16714018301 16714018302 16714018303 16714018401 16714018402 16714018403 16714018404 16714018405 18837012130 18837012230 18837022230 18837027830 18837030630 21695010130 21695010160 21695010190 21695010230 21695010260 21695010290 21695010330 21695010390 21695010430 21695010460 21695010490 21695015915 21695015930 21695016030 21695046590 21695055230 23490605901 23490605902 23490605903 23490606001 23490606002 23490694701 23490694702 23490694801 23490694802 23490694803 33261009002 33261009007 33261009010 33261009014 33261009020 33261009021 33261009028 33261009030 33261009060 33261009090 33261009110 33261009114 33261009121 33261009128 33261009130 33261009140 33261009160 33261009190 33261009230 33261009260 33261009290 33261009307 33261009314 33261009320 33261009321 33261009328 33261009330 33261009340 33261009360 33358028330 33358028360 33358028390 35356024630 35356089230 35356089260 35356089290 35356099430 35356099460 35356099490 42291057950 42291058050 42291058130 42858070303 42858070503 42858070703 43063017090 43547034703 43547034709 43547034711 43547034750 43547034803 43547034809 43547034811 43547034850 43547034903 43547034909 43547034911 43547034950 43547035003 43547035009 43547035011 43547035050 43547040903 45865044200 45865044230 45865044251 45865044260 45865044290 45865046300 45865046330 45865046351 45865046360 45865046390 47463057130 47463057145 47463057160 49884087605 49884087611 49884087701 49884087705 49884087711 49884087805 49884087811 49884087905 49884087911 49999059730 49999060115 49999060130 49999061330 49999063130 49999063160 49999063200 49999063230 49999063260 49999078030 49999082830 50090084900 50090084901 50090084902 50090085000 50090085001 50090085002 50090092100 50090092101 50090092300 50090092301 50090129600 50090129601 50090129700 50090129701 50090277800 50090325600 50090325601 50090483800 50090526300 50090526301 50268064011 50268064015 50268064111 50268064115 50268064211 50268064215 50268064311 50268064315 50436393303 51079074401 51079077401 51079077420 51079077501 51079077520 51079082463 51079082563 52343007330 52343007399 52343007430 52343007499 52343007530 52343007590 52343007630 52343007690 52817014050 52817014090 52817014100 52817014190 52817014250 52817014290 52817014350 52817014390 52959036012 52959036015 52959036020 52959036030 52959036060 52959063930 52959077530 52959077550 52959077560 52959077590 52959077630 52959077660 52959079230 53217017730 53217017760 53217017790 54458098810 54458098816 54458098904 54458098910 54458098916 54458099010 54458099016 54569381000 54569381001 54569478700 54569490100 54569490101 54569554100 54569554101 54569554102 54569559800 54569568200 54569568201 54569589800 54569631800 54569631801 54569860900 54569908600 54766020101 54766020201 54766020301 54766020401 54766090753 54868181900 54868297600 54868297602 54868297603 54868352600 54868352601 54868396200 54868406500 54868479100 54868481700 54868481701 54868481702 54868493700 54868493701 54868493702 54868493703 54868493704 54868493800 54868493801 54868508000 54868534700 54868536500 54868617800 55045254608 55045317108 55045317208 55045317209 55048057130 55048057145 55048057160 55048057230 55048057330 55048057390 55048060830 55154475300 55154508700 55154664000 55154799900 55175271503 55289003745 55289003790 55289005345 55289005390 55289021630 55289097230 55289097260 55289097290 55700004060 55700037930 55700037960 55700037990 55700050330 55887021330 55887021430 55887034630 55887039430 55887039460 55887039490 55887049030 55887049060 55887049082 55887049090 55887051120 55887051130 55887051160 55887051182 55887051190 55887054930 55887054960 55887054990 57664042113 57664042183 57664042199 57664042213 57664042218 57664042283 57664042299 57664042413 57664042483 57664042499 57664042513 57664042583 57664042599 57866004508 58016048500 58016048502 58016048510 58016048512 58016048515 58016048520 58016048525 58016048530 58016048540 58016048550 58016048560 58016048570 58016048580 58016048590 58016066100 58016066130 58016066160 58016066190 58016073100 58016073130 58016073160 58016073190 58016074900 58016074902 58016074930 58016074960 58016074990 58016076100 58016076130 58016076160 58016076190 58016080600 58016080630 58016080660 58016080690 58016081700 58016081702 58016081703 58016081730 58016081760 58016081790 58016081800 58016081802 58016081803 58016081830 58016081860 58016081890 58016081899 58016089700 58016089730 58016089760 58016089790 58016090700 58016090730 58016090760 58016090790 58864037215 58864037230 58864062815 58864071615 58864071630 58864074115 58864074130 59746045710 59746045730 59746045790 59746045810 59746045830 59746045890 59746045910 59746045930 59746045990 59746046010 59746046030 59746046090 59762180801 59762180802 59762180803 59762181001 59762181002 59762181003 59762181004 59762181201 59762181202 59762181203 59762181501 59762181502 59762181503 60346099330 60429073401 60429073405 60429073410 60429073430 60429073490 60429073501 60429073505 60429073510 60429073530 60429073590 60429073601 60429073605 60429073610 60429073630 60429073690 60429073701 60429073705 60429073710 60429073715 60429073730 60429073745 60429073790 60505008300 60505008301 60505008302 60505008304 60505008401 60505008402 60505008404 60505009701 60505009702 60505009704 60505010101 60505010102 60505010104 60505010107 60505037401 60505040205 60505131603 60505131703 60505131803 60505366303 60505366403 60505366503 60505366603 60505366803 60505366903 60505367003 60505367303 60505367403 60505367503 60505437703 60505437803 60505437903 60505451703 60505451803 60505451903 60505452003 60760009890 60760009990 60760039890 60760050190 61919018130 61919018160 61919018190 61919032230 61919051230 61919051330 61919059630 61919065830 61919065860 61919065890 62037084530 62037084601 62037084610 62037084630 62037084730 62037084830 62135042530 62135042630 62135042730 62175047032 62175047041 62175047132 62175047141 62175047232 62175047241 63187019030 63187043430 63187092730 63187092790 63629184001 63629184002 63629320501 63629320502 63629320503 63629334801 63629334802 63629334803 63629334804 63629461501 63629461502 63629461503 63629461504 63629461505 63629852701 63629949801 63629949901 63629950001 63672201001 63672202001 63672203001 63672204001 63739040710 63739040810 63739088810 63739088841 63739096310 63739096341 63874053801 63874053810 63874053814 63874053815 63874053820 63874053830 63874053860 63874100303 63874112506 63874115103 65862015430 65862015505 65862015530 65862015599 65862015630 65862015699 65862015730 65862015799 66105011603 66105011703 66105011810 66267072130 66336079930 66336079960 66336079990 68071003430 68084004401 68084004405 68084004411 68084004430 68084004485 68084004490 68084004500 68084004501 68084004505 68084004511 68084004530 68084004585 68084004590 68084004601 68084004605 68084004611 68084004630 68084004685 68084004690 68084004701 68084004705 68084004711 68084004730 68084004785 68084004790 68115026730 68115029730 68115047730 68115047760 68115047790 68115048360 68115065630 68115073800 68115073830 68115074000 68115074030 68115075130 68115080130 68115091330 68115091630 68115091930 68180064506 68180064606 68180064706 68258700703 68258700803 68258700903 68258798809 68382000101 68382000105 68382000106 68382000110 68382000116 68382009701 68382009705 68382009706 68382009710 68382009716 68382009801 68382009805 68382009806 68382009810 68382009816 68382009901 68382009905 68382009906 68382009910 68382009916 68387011230 68788079709 68788741001 68788741002 68788741003 68788741006 68788741008 68788741009 68788767701 68788767703 68788767706 68788767708 68788767709 68788907403 68788907406 68968201001 68968202001 68968203001 68968204001 68968907503 68968907507 69367033530 69367033630 69367033730 69584067103 69584067109 69584067150 69584067190 69584067203 69584067209 69584067210 69584067250 69584067290 69584067303 69584067309 69584067350 69584067390 69584067403 69584067409 69584067450 69584067490 70934010730 70934066130 70954031910 71205019830 71205019890 71205052730 71335032101 71335032102 71335032103 71335032104 71335060301 71335060302 71335060303 71335067502 71335075001 71335150601 |
| Sertraline | 49005001 49490030 49490041 49490050 49490066 49490073 49490094 49491030 49491041 49491050 49491066 49491073 49491094 49494023 49496030 49496050 54002213 54002313 54002328 54002413 54002428 54008246 93717510 93717556 93717610 93717656 93717710 93717756 143958005 143958009 143958030 143958105 143958109 143958130 143958209 143958230 143965405 143965409 143965430 143965505 143965509 143965530 143965609 143965630 172567210 172567280 172567310 172567410 185005730 185015330 185026530 228272103 228272109 228272150 228272203 228272209 228272290 228272303 228272309 228272396 378418601 378418605 378418693 378418701 378418705 378418793 378418801 378418805 378418893 378801101 378801105 378812101 378812105 378812701 378812705 403432101 403432130 403472130 591323819 591323830 591323910 591323919 591323930 591324010 591324019 591324030 904586661 904586761 904586861 904608761 904608861 904608961 904633161 904633261 904633361 904692461 904692561 904692661 10135069461 12634090471 13411015201 13411015203 13411015206 13411015209 13411015215 13411015301 13411015303 13411015306 13411015309 13411015315 13668000401 13668000405 13668000410 13668000430 13668000450 13668000490 13668000501 13668000505 13668000510 13668000530 13668000550 13668000590 13668000601 13668000605 13668000610 13668000630 13668000650 13668000690 16252053330 16252053350 16252053430 16252053450 16252053490 16252053530 16252053550 16252053590 16590024930 16590024960 16590024990 16590025030 16590025060 16590025090 16590025130 16590025160 16590025190 16590041610 16590041615 16590041630 16590041660 16590041672 16590041690 16590045715 16590045728 16590045730 16590045745 16590045756 16590045760 16590045772 16590045790 16590070030 16590070082 16714060101 16714060102 16714061101 16714061104 16714061105 16714061106 16714061201 16714061204 16714061205 16714061206 16714061301 16714061304 16714061305 16714061306 16729021510 16729021515 16729021516 16729021615 16729021616 16729021715 16729021716 18837018530 18837018560 18837018590 18837018660 18837018730 18837024830 18837024860 18837024890 18837024930 18837024960 18837024990 21695016430 21695016530 21695016560 21695016590 21695016630 21695016660 21695016690 23490626301 23490626302 23490626303 23490626401 23490626402 23490626403 23490626404 23490705001 23490705002 31722014505 31722014530 31722014590 31722014605 31722014630 31722014690 31722014705 31722014730 31722014790 31722021205 31722021230 31722021290 31722021305 31722021330 31722021390 31722021405 31722021430 31722021490 33261017000 33261017007 33261017014 33261017021 33261017028 33261017030 33261017060 33261017090 33261017114 33261017115 33261017128 33261017130 33261017160 33261034607 33261034610 33261034614 33261034620 33261034621 33261034628 33261034630 33261034660 33261034690 33358032130 33358032160 33358032190 33358032230 33358032260 33358032290 33358032330 33358032360 33358032390 35356003330 35356066830 35356066860 35356066890 35356066930 35356083230 35356083260 35356083290 43063003301 43063003330 43063003360 43063003390 43063041130 43063041160 43063041190 43063041390 43063075430 43063075460 43063075490 43063085530 43063085560 43063085590 45865052430 45865052451 45865052460 45865052490 46144010801 46144010805 47463077030 47463077060 47463077090 47463077130 47463077230 47463077260 47463077290 49999029215 49999029230 49999037500 49999037515 49999037530 49999077630 49999077650 49999086030 49999086060 49999086090 49999086130 49999086160 49999086190 50090098900 50090098901 50090098902 50090207100 50090207101 50090207102 50090208000 50090208001 50090208002 50090277900 50090297600 50090297601 50090308902 50090311200 50090315300 50090320300 50090320301 50090320302 50090321700 50090394500 50090394501 50090394700 50090394701 50111093010 50111093101 50111093102 50111093110 50111093201 50111093202 50111093210 50436352001 50436630401 50436630501 50436630601 51079014901 51079014920 51079015001 51079015020 51079015101 51079015120 51079076201 51079076220 51079076301 51079076320 51079076401 51079076420 51552137308 51655035226 51655066224 51655097945 51927422600 51927474600 52427066430 52427067230 52959036100 52959036114 52959036130 52959036160 52959078130 52959078160 52959078730 52959087230 52959087238 52959087260 52959087530 52959087560 52959087590 52959087600 52959087630 52959087660 53217024230 53217024260 53217024290 53217025715 53217025730 53217025760 54458091302 54458092405 54458094405 54458094410 54458094510 54458094710 54569357500 54569357501 54569357502 54569357503 54569372400 54569372401 54569372402 54569372403 54569372404 54569372405 54569452900 54569452901 54569452902 54569581800 54569581801 54569581802 54569581900 54569581901 54569581902 54569619200 54569857900 54569907600 54569911800 54868219200 54868219201 54868219203 54868219204 54868219205 54868219206 54868219207 54868219208 54868263700 54868263701 54868263703 54868263704 54868263705 54868263706 54868263707 54868263708 54868437200 54868437201 54868437202 54868563800 54868563801 54868563802 54868563803 54868563804 54868563805 54868563806 54868563807 54868563808 54868563900 54868563901 54868563902 54868563903 54868563904 54868563905 54868563906 54868565800 55045220801 55045220803 55045220807 55045220808 55045222400 55045222402 55045222407 55045222408 55045338608 55045356201 55045356601 55045356801 55048077030 55048077060 55048077090 55048077130 55048077230 55048077260 55048077290 55111017330 55111017405 55111017430 55111017505 55111017530 55154052700 55154468700 55154469200 55175271403 55175271404 55175271406 55175271608 55289029114 55289029130 55289029160 55289037830 55289038130 55289038145 55289038160 55289038190 55289040930 55289040960 55289055015 55289055030 55700007890 55700022230 55700022530 55700022560 55700022590 55700034030 55700034060 55700034090 55700036200 55700036230 55700036260 55700036290 55700056230 55700056290 55700091090 55700094830 55700094890 55700097790 55887016015 55887016030 55887016060 55887016090 55887016830 55887048730 55887048760 55887048782 55887048790 55887051920 55887051930 55887051960 55887051982 55887051990 55887092530 55887092560 55887092582 55887092590 55887096715 55887096730 55887096760 55887096790 57866630500 57866630501 58016000900 58016000930 58016000960 58016000990 58016001000 58016001002 58016001030 58016001060 58016001090 58016001100 58016001130 58016001160 58016001190 58016036600 58016036630 58016036660 58016036690 58016066400 58016066430 58016066460 58016066490 58016066800 58016066830 58016066860 58016066890 58864036615 58864036630 58864062715 58864062730 58864070730 58864089330 59762006701 59762490001 59762490002 59762490003 59762490004 59762490005 59762491001 59762491002 59762491003 59762491004 59762491005 59762494001 59762496001 59762516001 59762516002 59762516003 59762634701 59762644301 59762644302 59762644303 60346051612 60346051630 60346051650 60346070715 60346070730 60505018003 60505018008 60505018103 60505018108 60505018203 60505018208 60687023101 60687023111 60687024201 60687024211 60687025301 60687025311 60760009190 60760030830 60760030960 60760030990 60760031030 60760031090 60760061130 60760061290 60760073730 60760073790 60760073890 60760083430 60760083490 60760083530 60760083590 61919001460 61919008620 61919008630 61919008660 61919008690 61919034530 61919034560 61919034590 61919041630 61919045730 61919045760 61919045790 63187005530 63187021230 63187047830 63187047890 63187076730 63187076760 63187079530 63187079590 63304016430 63304016501 63304016505 63304016530 63304016601 63304016605 63304016630 63304084005 63629328901 63629328902 63629328903 63629328904 63629330901 63629330902 63629330903 63629330904 63629330905 63629331301 63629331302 63629331303 63629331304 63629877401 63874055501 63874055510 63874055514 63874055515 63874055520 63874055530 63874055560 63874059601 63874059610 63874059614 63874059615 63874059620 63874059630 63874059660 64679075201 64679075204 64679075207 64679075301 64679075304 64679075307 64980040906 65862001101 65862001105 65862001130 65862001190 65862001201 65862001205 65862001230 65862001290 65862001301 65862001305 65862001330 65862001390 66105056010 66105056110 67801020410 67801020510 68071054828 68071054856 68071054860 68071070228 68071070260 68071131700 68071131800 68084018001 68084018011 68084018101 68084018111 68084018201 68084018211 68115036500 68115036515 68115036530 68115036545 68115036560 68115036600 68115036615 68115036630 68115036660 68115076530 68115076550 68180035103 68180035106 68180035109 68180035201 68180035202 68180035205 68180035206 68180035209 68180035301 68180035302 68180035305 68180035306 68180035309 68258701003 68258701009 68258705401 68258705403 68258705406 68258705408 68258705409 68258705509 68258708603 68258708606 68258708609 68387011930 68645042454 68645042554 68645042654 68645048770 68645048870 68645048970 68645049801 68645049901 68645050001 68645052101 68645052154 68645052170 68645052201 68645052254 68645052270 68645052301 68645052354 68645052370 68645058954 68788642903 68788642909 68788643703 68788643709 68788683501 68788683503 68788683506 68788683508 68788683509 68788792801 68788792803 68788792806 68788792809 68788810901 68788810903 68788810906 68788810909 68788821003 68788821006 68788821009 69097083302 69097083305 69097083312 69097083402 69097083412 69097083502 69097083512 70934001930 70934001990 70934016990 70934018830 70934018890 71093014311 71205002830 71205002890 71205004730 71205004760 71205004790 71205020530 71205020560 71205020590 71205034330 71205045530 71205045590 71335021001 71335040301 71335040302 71335040303 71335040304 71335041501 71335071801 71335071802 71335071803 71335071804 71335071805 71335071806 71335071807 71335071808 71335071809 71335080801 71335089601 71335089602 71335089603 72189012230 72189012260 72789001330 72789001360 72789001390 72789008860 72789009230 72789009260 72789009290 72789027301 76282021201 76282021205 76282021218 76282021230 76282021260 76282021290 76282021301 76282021305 76282021318 76282021330 76282021360 76282021390 76282021401 76282021405 76282021418 76282021430 76282021460 76282021490 |

**Table S7.** Procedure codes related to the psychotherapy used for PTSD management

| **CPT code** | **Description** |
| --- | --- |
| Individual psychotherapy | 90832 90833 90834 90836 90837 90839 90840 |
| Family psychotherapy | 90846 90847 90849 |
| Group psychotherapy | 90853 |
| Interactive psychotherapy | 90785 |
| Psychotherapy with pharmacological management | 90863 |

**Table S8.** Demographic characteristics of the matched population

|  | **Total Sample**  **(N=5076)** | **Baseline PTSD (N=1714)** | **PTSD without Comorbidities (N=1681)** | **PTSD with Comorbidities (N=1681)** | **P-value*** |
| --- | --- | --- | --- | --- | --- |
| Age, mean (SD) | 40.9 (12.1) | 41.0 (12.1) | 40.8 (12.0) | 40.8 (12.4) | 0.977 |
| **Gender, n (%)** |  |  |  |  |  |
| Women | 2725 (53.7) | 907 (52.9) | 911 (54.2) | 907 (54.0) | 0.729 |
| Men | 2351 (46.3) | 807 (47.1) | 770 (45.8) | 774 (46.0) | 0.729 |
| **Health Plan, n (%)** |  |  |  |  |  |
| Preferred Provider Organization | 2736 (53.7) | 908 (53.0) | 903 (53.7) | 915 (54.4) | 0.696 |
| Health Maintenance Organization | 972 (19.1) | 333 (19.4) | 313 (18.6) | 326 (19.4) | 0.796 |
| Consumer-Driven Health Plan | 630 (12.4) | 214 (12.5) | 216 (12.8) | 200 (11.9) | 0.700 |
| High Deductible Health Plan | 507 (10.0) | 170 (9.9) | 167 (9.9) | 170 (10.1) | 0.978 |
| Non-Capitated Point-of-Service | 162 (3.2) | 60 (3.5) | 52 (3.1) | 50 (3.0) | 0.657 |
| Comprehensive Plan | 63 (1.2) | 23 (1.3) | 24 (1.4) | 16 (1.0) | 0.413 |
| Unknown | 10 (0.2) | 4 (0.2) | 4 (0.2) | 2 (0.1) | 0.677 |
| Exclusive Provider Organization | 4 (0.1) | 2 (0.1) | 1 (0.1) | 1 (0.1) | 0.790 |
| Point-of-Service with Capitation | 2 (0.0) | 0 | 1 (0.1) | 1 (0.1) | 0.600 |
| **Region, n (%)** |  |  |  |  |  |
| South | 2511 (49.5) | 855 (49.9) | 841 (50.0) | 815 (48.5) | 0.612 |
| West | 1133 (22.3) | 380 (22.2) | 370 (22.0) | 383 (22.8) | 0.851 |
| North central | 944 (18.6) | 317 (18.5) | 310 (18.4) | 317 (18.9) | 0.944 |
| Northeast | 488 (9.6) | 162 (9.5) | 160 (9.5) | 166 (9.9) | 0.904 |

**Chi-square test was performed for categorical variables and ANOVA (Tamhane's T2 test) for continuous variables*

**Table S9.** Clinical characteristics of the matched population

|  | **Total Sample**  **(N=5,076)** | **Baseline PTSD (N=1,714)** | **PTSD without Comorbidities (N=1,681)** | **PTSD with Comorbidities (N=1,681)** | **P-value*** |
| --- | --- | --- | --- | --- | --- |
| **Charlson Comorbidity score** |  |  |  |  |  |
| 0 | 4113 (81.0) | 1395 (81.4) | 1357 (80.7) | 1361 (81.0) | 0.883 |
| 1 | 718 (14.1) | 239 (13.9) | 245 (14.6) | 234 (13.9) | 0.826 |
| 2 | 111 (2.2) | 37 (2.2) | 38 (2.3) | 36 (2.1) | 0.968 |
| 3 | 100 (2.0) | 32 (1.9) | 30 (1.8) | 38 (2.3) | 0.569 |
| 4+ | 34 (0.7) | 11 (0.6) | 11 (0.7) | 12 (0.7) | 0.963 |
| Charlson Comorbidity Index, mean (SD) | 0.3 (0.7) | 0.3 (0.7) | 0.3 (0.7) | 0.3 (0.7) | 0.906 |
| **Charlson Comorbidity Index Components** |  |  |  |  |  |
| Chronic pulmonary disease | 389 (7.7) | 129 (7.5) | 137 (8.1) | 123 (7.3) | 0.640 |
| Diabetes without chronic complication | 261 (5.1) | 84 (4.9) | 88 (5.2) | 89 (5.3) | 0.855 |
| Mild liver disease | 139 (2.7) | 50 (2.9) | 43 (2.6) | 46 (2.7) | 0.814 |
| Diabetes with chronic complication | 108 (2.1) | 37 (2.2) | 33 (2.0) | 38 (2.3) | 0.831 |
| Renal disease | 78 (1.5) | 27 (1.6) | 24 (1.4) | 27 (1.6) | 0.904 |
| Cerebrovascular disease | 68 (1.3) | 16 (0.9) | 27 (1.6) | 25 (1.5) | 0.190 |
| Rheumatic disease | 52 (1.0) | 14 (0.8) | 17 (1.0) | 21 (1.2) | 0.456 |
| Peripheral vascular disease | 24 (0.5) | 11 (0.6) | 6 (0.4) | 7 (0.4) | 0.442 |
| Peptic ulcer disease | 22 (0.4) | 9 (0.5) | 6 (0.4) | 7 (0.4) | 0.751 |
| Congestive heart failure | 18 (0.4) | 6 (0.4) | 7 (0.4) | 5 (0.3) | 0.844 |
| Myocardial infarction | 15 (0.3) | 8 (0.5) | 3 (0.2) | 4 (0.2) | 0.262 |
| Dementia | 8 (0.2) | 3 (0.2) | 2 (0.1) | 3 (0.2) | 0.887 |
| AIDS/HIV | 3 (0.1) | 0 | 2 (0.1) | 1 (0.1) | 0.362 |
| Hemiplegia or paraplegia | 7 (0.1) | 1 (0.1) | 1 (0.1) | 5 (0.3) | 0.098 |
| Moderate or severe liver disease | 2 (0.0) | 1 (0.1) | 0 | 1 (0.1) | 0.609 |
| Malignancy | 0 | 0 | 0 | 0 | - |
| Metastatic solid tumor | 0 | 0 | 0 | 0 | - |

**Chi-square test was performed for categorical variables and ANOVA (Tamhane's T2 test) for continuous variables*

**Table S10.** Healthcare costs of psychotherapy sessions among the matched sample of patients with PTSD during the 24-month post-index period

| **Mean Cost of Psychotherapy Sessions ($), mean (SD)** | **Payer Perspective Costs** | **Total Healthcare Costs** |
| --- | --- | --- |
| **Individual Psychotherapy Session** | | |
| 30-min psychotherapy | $60 (55) | $73 (65) |
| Standard 30-min psychotherapy | $67 (80) | $90 (106) |
| 30-min add-on psychotherapy | $57 (42) | $67 (39) |
| 30-min add-on psychotherapy for crisis | $87 (83) | $98 (77) |
| 45-min psychotherapy | $76 (98) | $100 (98) |
| Standard 45-min psychotherapy | $74 (101) | $99 (101) |
| 45-min add-on psychotherapy | $95 (59) | $114 (51) |
| 60-min psychotherapy | $84 (54) | $109 (48) |
| Standard 60-min psychotherapy | $84 (53) | $109 (47) |
| 60-min add-on psychotherapy | $84 (72) | $96 (70) |
| 60-min psychotherapy for crisis | $129 (126) | $150 (117) |
| 90-min psychotherapy | $165 (159) | $221 (148) |
| 120-min psychotherapy | $186 (156) | $239 (174) |
| **Family Psychotherapy Session** | $84 (73) | $109 (71) |
| **Group Psychotherapy Session** | $198 (432) | $222 (491) |
| **Add-on session with Interactive Complexity Psychiatry Service** | $9 (16) | $11 (17) |
| **Add-on session with Pharmacological Management** | $26 (25) | $45 (12) |

***Note:*** *The mean healthcare costs of 90-min and 120-min psychotherapy procedures were calculated per patient who had 90- and 120-minute total duration of all psychotherapy procedures provided on the same date (e.g., 60-min standard psychotherapy plus 30-min add-on session)*

**Table S11.** Demographic characteristics of patients with PTSD and SUD/AUD diagnosis

|  | **Total Sample**  **(N=3776)** | **Baseline PTSD (N=85)** | **PTSD without Comorbidities (N=537)** | **PTSD with Comorbidities (N=3154)** | **P-value*** |
| --- | --- | --- | --- | --- | --- |
| Age, mean (SD) | 35.2 (13.4) | 38.7 (13.9) | 38.3 (13.1) | 34.6 (13.3) | ≤0.024 ^1, 2^ |
| **Gender, n (%)** |  |  |  |  |  |
| Women | 2246 (59.5) | 28 (32.9) | 223 (41.5) | 1995 (63.3) | <0.001 ^1, 2^ |
| Men | 1530 (40.5) | 57 (67.1) | 314 (58.5) | 1159 (36.7) | <0.001 ^1, 2^ |
| **Health Plan, n (%)** |  |  |  |  |  |
| Preferred Provider Organization | 1850 (49.0) | 40 (47.1) | 256 (47.7) | 1554 (49.3) | 0.741 |
| Health Maintenance Organization | 637 (16.9) | 18 (21.2) | 89 (16.6) | 530 (16.8) | 0.558 |
| Consumer-Driven Health Plan | 494 (13.1) | 9 (10.6) | 67 (12.5) | 418 (13.3) | 0.698 |
| High Deductible Health Plan | 400 (10.6) | 8 (9.4) | 66 (12.3) | 326 (10.3) | 0.372 |
| Non-Capitated Point-of-Service | 195 (5.2) | 3 (3.5) | 29 (5.4) | 163 (5.2) | 0.769 |
| Comprehensive Plan | 137 (3.6) | 5 (5.9) | 18 (3.4) | 114 (3.6) | 0.508 |
| Unknown | 38 (1.0) | 1 (1.2) | 7 (1.3) | 30 (1.0) | 0.742 |
| Exclusive Provider Organization | 16 (0.4) | 0 | 3 (0.6) | 13 (0.4) | 0.740 |
| Point-of-Service with Capitation | 9 (0.2) | 1 (1.2) | 2 (0.4) | 6 (0.2) | 0.145 |
| **Region, n (%)** |  |  |  |  |  |
| South | 1525 (40.4) | 38 (44.7) | 225 (41.9) | 1262 (40.0) | 0.509 |
| North central | 942 (24.9) | 22 (25.9) | 130 (24.2) | 790 (25.0) | 0.899 |
| West | 759 (20.1) | 17 (20.0) | 119 (22.2) | 623 (19.8) | 0.437 |
| Northeast | 549 (14.5) | 8 (9.4) | 63 (11.7) | 478 (15.2) | 0.038 ^2^ |
| Unknown | 1 (0.0) | 0 | 0 | 1 (0.0) | 0.906 |

**Chi-square test was performed for categorical variables and ANOVA (Tamhane's T2 test) for continuous variables*

*^1^ Baseline PTSD vs. PTSD without Comorbidities, p<0.05*

*^2^ Baseline PTSD vs. PTSD with Comorbidities, p<0.05*

**Table S12.** Clinical characteristics of patients with PTSD and SUD/AUD diagnosis

|  | **Total Sample**  **(N=3,776)** | **Baseline PTSD (N=85)** | **PTSD without Comorbidities (N=537)** | **PTSD with Comorbidities (N=3154)** | **P-value*** |
| --- | --- | --- | --- | --- | --- |
| **Charlson Comorbidity Index** |  |  |  |  |  |
| 0 | 2684 (71.1) | 57 (67.1) | 393 (73.2) | 2234 (70.8) | 0.383 |
| 1 | 610 (16.2) | 17 (20.0) | 78 (14.5) | 515 (16.3) | 0.359 |
| 2 | 201 (5.3) | 2 (2.4) | 25 (4.7) | 174 (5.5) | 0.333 |
| 3 | 136 (3.6) | 2 (2.4) | 23 (4.3) | 111 (3.5) | 0.560 |
| 4+ | 145 (3.8) | 7 (8.2) | 18 (3.4) | 120 (3.8) | ≤0.038 ^1, 2^ |
| Charlson Comorbidity Index, mean (SD) | 0.6 (1.2) | 0.8 (1.6) | 0.5 (1.1) | 0.6 (1.2) | 0.426 |
| **Charlson Comorbidity Index Components** |  |  |  |  |  |
| Chronic pulmonary disease | 460 (12.2) | 10 (11.8) | 50 (9.3) | 400 (12.7) | 0.027 ^3^ |
| Mild liver disease | 240 (6.4) | 5 (5.9) | 39 (7.3) | 196 (6.2) | 0.644 |
| Diabetes without chronic complication | 223 (5.9) | 9 (10.6) | 32 (6.0) | 182 (5.8) | 0.177 |
| Renal disease | 199 (5.3) | 6 (7.1) | 25 (4.7) | 168 (5.3) | 0.615 |
| Diabetes with chronic complication | 135 (3.6) | 3 (3.5) | 19 (3.5) | 113 (3.6) | 0.998 |
| Cerebrovascular disease | 73 (1.9) | 3 (3.5) | 9 (1.7) | 61 (1.9) | 0.514 |
| Rheumatic disease | 54 (1.4) | 3 (3.5) | 3 (0.6) | 48 (1.5) | 0.036 ^1^ |
| Peptic ulcer disease | 41 (1.1) | 1 (1.2) | 6 (1.1) | 34 (1.1) | 0.993 |
| AIDS/HIV | 33 (0.9) | 1 (1.2) | 3 (0.6) | 29 (0.9) | 0.677 |
| Congestive heart failure | 34 (0.9) | 3 (3.5) | 3 (0.6) | 28 (0.9) | ≤0.046 ^1, 2^ |
| Peripheral vascular disease | 31 (0.8) | 2 (2.4) | 4 (0.7) | 25 (0.8) | 0.284 |
| Hemiplegia or paraplegia | 30 (0.8) | 1 (1.2) | 3 (0.6) | 26 (0.8) | 0.751 |
| Myocardial infarction | 29 (0.8) | 1 (1.2) | 7 (1.3) | 21 (0.7) | 0.267 |
| Dementia | 19 (0.5) | 0 | 3 (0.6) | 16 (0.5) | 0.793 |
| Moderate or severe liver disease | 19 (0.5) | 1 (1.2) | 5 (0.9) | 13 (0.4) | 0.196 |
| Malignancy | 0 | 0 | 0 | 0 | - |
| Metastatic solid tumor | 0 | 0 | 0 | 0 | - |

**Chi-square test was performed for categorical variables and ANOVA (Tamhane's T2 test) for continuous variables*

*^1^ Baseline PTSD vs. PTSD without Comorbidities, p<0.05*

*^2^ Baseline PTSD vs. PTSD with Comorbidities, p<0.05*

*^3^ PTSD without Comorbidities vs. PTSD with Comorbidities, p<0.05*

**Table S13.** Healthcare costs of psychotherapy sessions among patients with PTSD and SUD/AUD during the 24-month post-index period

| **PTSD Treatments** | **Payer Perspective Costs** | **Total Healthcare Costs** |
| --- | --- | --- |
| **Mean Cost of Individual Psychotherapy Session ($), mean (SD)** | | |
| 30-min psychotherapy | $64 (100) | $76 (112) |
| Standard 30-min psychotherapy | $73 (169) | $92 (192) |
| 30-min add-on psychotherapy | $60 (40) | $69 (37) |
| 30-min add-on psychotherapy for crisis | $61 (49) | $68 (49) |
| 45-min psychotherapy | $87 (77) | $108 (90) |
| Standard 45-min psychotherapy | $86 (78) | $107 (91) |
| 45-min add-on psychotherapy | $102 (72) | $118 (68) |
| 60-min psychotherapy | $92 (71) | $115 (69) |
| Standard 60-min psychotherapy | $92 (70) | $115 (68) |
| 60-min add-on psychotherapy | $98 (68) | $113 (64) |
| 60-min psychotherapy for crisis | $115 (127) | $135 (135) |
| 90-min psychotherapy | $164 (142) | $202 (155) |
| 120-min psychotherapy | $220 (178) | $273 (196) |
| **Family Psychotherapy Session** | $109 (113) | $134 (117) |
| **Group Psychotherapy Session** | $171 (370) | $190 (403) |
| **Add-on session with Interactive Complexity Psychiatry Service** | $17 (34) | $18 (34) |
| **Add-on session with Pharmacological Management** | $154 (288) | $170 (299) |

***Note:*** *The mean healthcare costs of 90-min and 120-min psychotherapy procedures were calculated per patient who had 90- or 120-minute total duration of all psychotherapy procedures provided on the same date (e.g., 60-min standard psychotherapy plus 30-min add-on session)*
